# Evaluation and identification of wild lentil accessions for enhancing genetic gains of cultivated varieties

**DOI:** 10.1371/journal.pone.0229554

**Published:** 2020-03-03

**Authors:** Mohar Singh, Sandeep Kumar, Ashwani Kumar Basandrai, Daisy Basandrai, Nikhil Malhotra, Deep Rattan Saxena, Dorin Gupta, Ashutosh Sarker, Kuldeep Singh

**Affiliations:** 1 Regional Station, National Bureau of Plant Genetic Resources, Shimla, India; 2 National Bureau of Plant Genetic Resources, Pusa, New Delhi, India; 3 CSK Himachal Pradesh Agriculture University, Palampur, India; 4 Rafi Ahmad Kidwai College of Agriculture, Sehore, India; 5 Faculty of Veterinary and Agricultural Sciences, The University of Melbourne, Melbourne, Australia; 6 South Asia and China Regional Programme, International Centre for Agricultural Research in Dry Areas, DPS Marg, Pusa, New Delhi, India; Louisiana State University College of Agriculture, UNITED STATES

## Abstract

Domesticated lentil has a relatively narrow genetic base globally and most released varieties are susceptible to severe biotic and abiotic stresses. The crop wild relatives could provide new traits of interest for tailoring novel germplasm and cultivated lentil improvement. The primary objective of this study was to evaluate wild lentil accessions for identification of economically viable agro-morphological traits and resistance against major biotic stresses. The study has revealed substantial variations in seed yield and its important component characters. Further, the diversity analysis of wild accessions showed two major clusters which were bifurcated into sub-clusters, thereby suggesting their wider genetic divergence. However, principal component analysis exhibited that seed yield plant^-1^, number of seeds plant^-1^, number of pods plant^-1^, harvest index and biological yield plant^-1^ contributed significantly to the total genetic variation assessed in wild lentil taxa. Moreover, some of the wild accessions collected from Syria and Turkey regions showed resistance against more than one disease indicating rich diversity of lentil genetic resources. The identification of most promising genotypes carrying resistance against major biotic stresses could be utilized in the cultivated or susceptible varieties of lentil for enhancing genetic gains. The study has also identified some trait specific accessions, which could also be taken into the consideration while planning distant hybridization in lentil.

## Introduction

Lentil is a self-pollinating true diploid (2n = 2x = 14) grain legume crop having genome size of 4063 Mb [1]. The cultivated lentil species (*Lens culinaris*) encompasses two groups, established on the basis of distinct morphological characters, the small-seeded (*microsperma*) and large-seeded (*macrosperma*) [2]. The production and productivity of lentil has increased from an average yield of 565 kg ha^-1^ in 1961–63 to 1153 kg ha^-1^ during 2016–2017 [3]. Despite the tremendous improvement, the current lentil yield is much below as compared to other pulses. In India, most of the lentil varieties which were developed through pure line selection showed low yield potential. In cultivated lentil varieties, the yield limiting factors are lack of seedling vigour, very high rate flower drop and low pod setting, lack of lodging resistance and exposure to major biotic and abiotic stresses [4]. The pedigree analysis of some important released varieties exhibited that a few donors have contributed substantially into the background of those varieties [5]. Therefore, to attain further breakthrough for enhancing genetic gains, new target traits are needed to be identified and introgressed into cultivated gene pool for widening the genetic base of cultigens. Crop wild relatives (CWRs) are an invaluable reservoir of productivity enhancement related characters having resilience to climate change and farming system, and are source of novel traits [6–7]. The global wild lentil germplasm introduced from the International Centre for Agricultural Research in Dry Areas (ICARDA), has been multiplied, characterized and evaluated against target characters [8]. The promising 96 wild lentil accessions selected from 405 global wild collections were validated under multilocational evaluation for identifying stable donors against the target traits. The present study was therefore, undertaken (1) to evaluate promising wild lentil accessions for various target agronomical characters and major biotic stresses and (2) to identify the potential accessions (gene sources) carrying important traits for enhancing genetic gains of cultivated gene pool.

## Materials and methods

### Plant materials

The experimental genetic material comprised of 96 wild lentil accessions (Table 1) selected from 405 global wild collections including 2 cultivated varieties [8] consisting of *L*. *orientalis* (24), *L*. *odemensis* (16), *L*. *tomentosus* (8), *L*. *nigricans* (17), *L*. *ervoides* (24), *L*. *lamottei* (5) and *L*. *culinaris* (cultivated) (2) along with two check varieties (Precoz [Argentina], and L830 [India]). These were evaluated under multi-location and multi-season performance for target characters viz. earliness, pod number, seed yield and resistance against rust, powdery mildew and Fusarium wilt under field and controlled screening conditions. The original identity of these accessions was same as mentioned by the Biodiversity and Integrated Gene Management Unit (BIGM) at ICARDA.

**Table 1:** List of taxa wise wild lentil accessions along with their country of origin.

S. No	Taxa/accession	Origin	S. No.	Taxa/accession	Origin
	***L*. *orientalis***			***L*. *lamottei***	
1	ILWL 7 (EC718234)	Turkey	50	ILWL 14 (EC718236)	France
2	ILWL 8 (EC718235)	Turkey	51	ILWL 15 (EC718238)	France
3	ILWL 75 (EC718456)	Israel	52	ILWL 29 (EC718251)	Spain
4	ILWL 89 (EC718470)	Turkey	53	ILWL 429 (EC718686)	Spain
5	ILWL 95 (EC718475)	Turkey	54	EC718692	France
6	ILWL 96 (EC718476)	Turkey		***L*. o*demensis***	
7	ILWL 101 (EC718479)	Turkey	55	ILWL 20 (EC718243)	Palestine
8	ILWL 117 (EC718488)	Syria	56	ILWL 35 (EC718276)	Turkey
9	ILWL 124 (EC718490)	Syria	57	ILWL 39 (EC718277)	Palestine
10	ILWL 181 (EC718491)	Syria	58	ILWL 165 (EC718281)	Syria
11	ILWL 227 (EC718513)	Syria	59	ILWL 166 (EC718282)	Syria
12	ILWL 230 (EC718515)	Syria	60	ILWL 167 (EC718283)	Syria
13	ILWL 243 (EC718519)	Syria	61	ILWL 203 (EC789113)	Syria
14	ILWL 246 (EC718521)	Syria	62	ILWL 235 (EC718291)	Syria
15	ILWL 278 (EC718529)	Turkey	63	ILWL 320 (EC718295)	Turkey
16	ILWL 330 (EC718548)	Syria	64	ILWL 357 (EC718297)	Syria
17	ILWL 343 (EC718554)	Syria	65	ILWL 361 (EC718298)	Syria
18	ILWL 344 (EC718555)	Syria	66	ILWL 409 (EC718301)	Syria
19	ILWL 349 (EC718560)	Syria	67	ILWL 436 (EC718302)	Turkey
20	ILWL 359 (EC718566)	Syria	68	ILWL 438 (EC718303)	Turkey
21	ILWL 384 (EC718585)	Tajikistan	69	EC718311	Israel
22	ILWL 443 (EC718596)	Turkey	70	EC718694	Syria
23	ILWL 476 (EC718605)	Turkey		***L*. *ervoides***	
24	ILWL 480 (EC718609)	Syria	71	EC718692	Spain
	***L*. *nigricans***		72	ILWL 43 (EC718316)	Croatia
25	ILWL 9 (EC718236)	Syria	73	ILWL 50 (EC718321)	Croatia
26	ILWL 14 (EC718267)	Syria	74	ILWL 51 (EC718322)	Montenegro
27	ILWL 15 (EC718238)	France	75	ILWL 58 (EC718329)	Turkey
28	ILWL16 (EC718239)	Alpes-Cote d'Azur	76	ILWL 60 (EC718331)	Turkey
29	ILWL 18 (EC718241)	France	77	ILWL 61 (EC718332)	Turkey
30	ILWL 19 (EC718242)	Spain	78	ILWL 63 (EC718333)	Turkey
31	ILWL 22 (EC718245)	Italy	79	ILWL 65 (EC718335)	Turkey
32	ILWL 31 (EC718254)	Spain	80	ILWL 66 (EC718336)	Turkey
33	ILWL 34 (EC718256)	Ukraine	81	ILWL 204 (EC718360)	Turkey
34	ILWL 37 (EC718257)	Turkey	82	ILWL 234 (EC718362)	Syria
35	ILWL 38 (EC718258)	Turkey	83	ILWL 269 (EC718370)	Turkey
36	ILWL 191 (EC718682)	Croatia	84	ILWL 276 (EC718374)	Turkey
37	ILWL 460 (EC718262)	Turkey	85	ILWL 292 (EC718378)	Turkey
38	EC718266	Italy	86	ILWL 321 (EC718383)	Turkey
39	EC718270	Croatia	87	ILWL 398 (EC718401)	Lebanon
40	EC718273	Spain	88	ILWL 401 (EC718404)	Lebanon
41	EC718275	Turkey	89	ILWL 408 (EC718407)	Syria
	***L*. *tomentosus***		90	ILWL 414 (EC718411)	Syria
42	ILWL 90 (EC718471)	Turkey	91	ILWL 418 (EC718413)	Syria
43	ILWL 195 (EC718682)	Syria	92	ILWL 441 (EC718417)	Turkey
44	ILWL 198 (EC718685)	Syria	93	ILWL 442 (EC718418)	Turkey
45	ILWL 199 (EC718686)	Syria	94	EC718439	Israel
46	ILWL 305 (EC728782)	Turkey		***L*. *culinaris***	
47	ILWL 308 (EC728782)	Turkey	95	ILL 8006	Syria
48	ILWL 480 (EC741251)	Syria	96	ILL 10829	Syria
49	EC718673	Syria			

### Agronomic evaluation

All 96 wild lentil accessions were evaluated under two agro-ecological locations viz. Experimental Farm of NBPGR Regional Station, Shimla (28^0^ 35´ N, 70^0^ 18´ E, altitude 2276 m amsl) during winter season of 2014–2015, 2015–2016 and 2016–2017; and CSKHPKV Mountain Agricultural Research and Extension Centre, Sangla (31^0^ 55´ N, 77^0^ 50´ E altitude 3450 m amsl) during summer season of 2015, 2016 and 2017. Each entry was replicated thrice in 3 m long row and 35 cm apart. The observations were recorded against important agro-morphological characters (days to flowering, days to maturity, plant height, number of branches plant^-1^, number of pods plant^-1^, number of seeds plant^-1^, number of seeds pod^-1^, 100-seed weight, seed yield plant^-1^, and biological yield plant^-1^) in both locations. Further, numerical data was subjected to statistical analysis using SAS software [9]. The coefficient of variation for various characters was determined using the formulae of Burton [10] and broad sense heritability was also calculated as per the method suggested by Lush [11]. The expected genetic advance was performed as per the procedure of Johnson et al. [12].

### Phenotypic diversity and association study

The phenotypic diversity analysis was carried out using quantitative data of 96 promising accessions of wild lentil. Euclidean distances were calculated using quantitative data which was used to establish relationship between interspecific accessions. DARwin 5.0 software of Perrier et al. [13] was used for hierarchical clustering of accessions using UPGMA mode based upon Jaccard’s coefficient. Correlation among various parameters was determined using SAS software [9].

### Screening against rust, powdery mildew and Fusarium wilt

#### Rust (*Uromyces fabae* (Grev.) Fuckel)

The experiment was conducted at CSKHPKV Research and Extension Centre, Dhaulakuan (30^0^ 49´ N, 77^0^ 59´ E, altitude 468 m amsl) during winter season of 2014–2015, 2015–2016 and 2016–2017. All the test accessions and rust susceptible check ‘var. L830’ were sown in plastic pots (20 cm diameter) filled with a mixture of top field soil and farm yard manure (10:1) in two replications. The pots with plants at pre-flowering stage (~60 days after sowing) of wild accessions were shifted in the field sown with rust susceptible varieties, K 75 and L 830 from India. The test accessions were frequently inoculated by spraying suspension of local isolate of *U*. *viciae fabae* (1x10^6^ spores/ml). The data were recorded using 1–9 rating scales at vegetative and reproductive stages as followed by Mayee and Datar [14].

#### Powdery mildew (*Erysiphe trifolii*)

Another experiment was undertaken at CSKHPKV Research and Extension Centre, Dhaulakuan during winter season of 2014–2015, 2015–2016 and 2016–2017. The plants of test accessions were raised in plastic pots in a mixture of soil and farmyard manure mixture (10:1) under green house conditions. The disease symptoms started developing at flower initiation stage and susceptible checks developed disease earlier and faster. The pots with heavily infected susceptible plants were transferred near the test accessions raised in the green house. The heavily infected plants were shaken with the wooden stick to dislodge the conidia which dispersed and infected the healthy plant parts of the test accessions as also suggested by Tiwari et al. [15]. The full infection of susceptible accessions was observed near green pod stage and scorings were done at different intervals using 1–9 rating scales [15].

#### Fusarium wilt (*Fusarium oxysporum* f. sp. *lentis* (Vasd. Srin.) Gord)

All 96 wild lentil accessions were screened against Fusarium wilt resistance at Rafi Ahmad Kidwai Agriculture College, Sehore, Madhya Pradesh (23^0^ 12´ N, 77^0^ 05´ E, altitude 502 m amsl) during winter season of 2014–2015 and 2015–2016. For screening of wild lentil accessions, protocol was standardized as per the method described by Bayaa and Erskine [16–19]. The performance of disease pressure was compared with the resistant (PL639) and susceptible (L-9-12) checks from India, which were repeated after 15 accessions of each replication. The data were taken using 1–9 rating scales as followed by Bayaa et al. [17, 19].

## Results

### Diversity for quantitative characters

**Fig 1:**
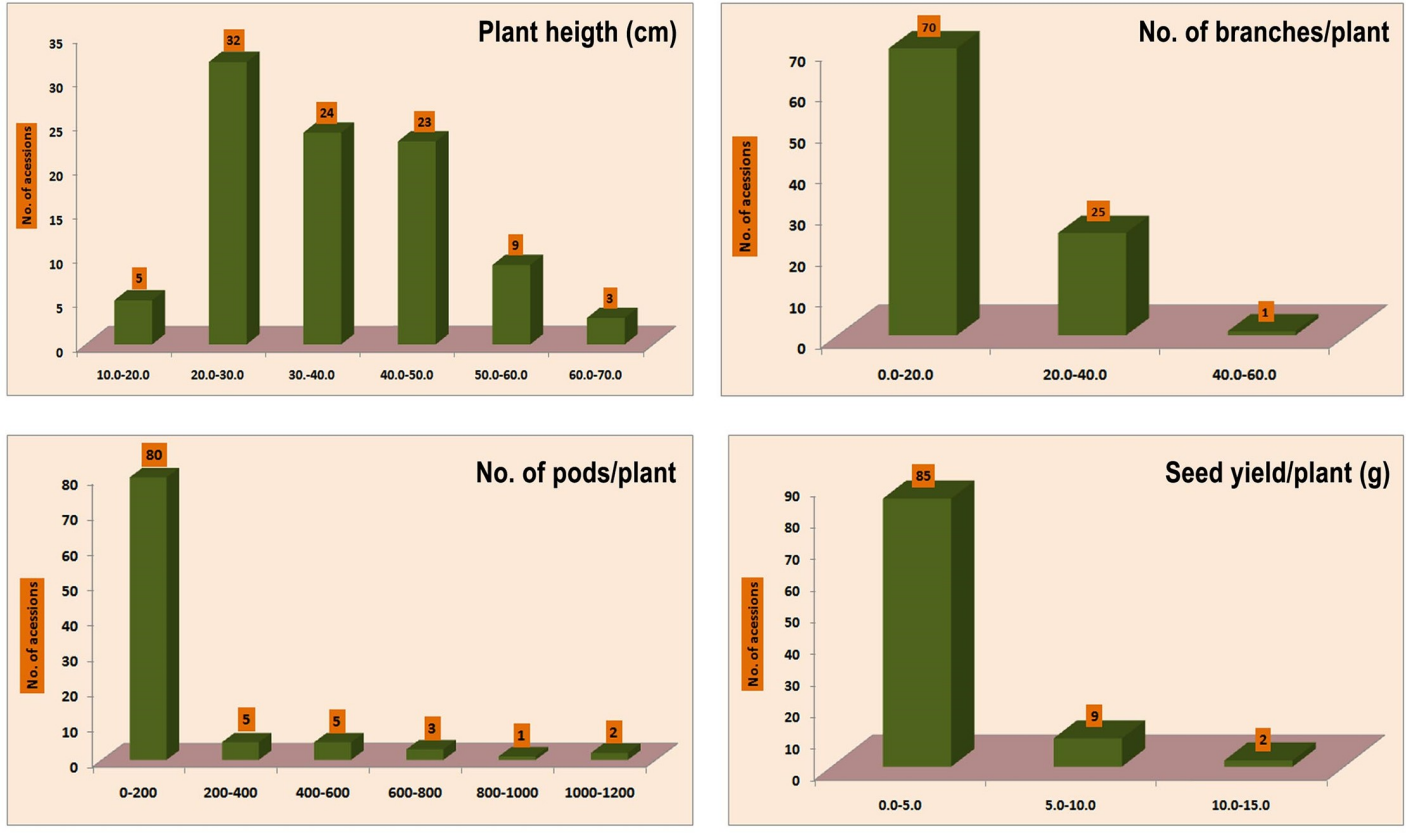
Diversity of wild lentil accessions for important quantitative traits.

The analysis of variance revealed that the wild lentil accessions differed significantly in terms of seed yield plant^-1^ and its important component characters (significant at *p* = 0.05) and also manifested by the range, mean and coefficient of variation for all the metric characters assessed. However, the frequency distribution of important quantitative characters depicted in Fig 1 exhibited that a wide range of variation was observed in plant height, number of branches plant^-1^, number of pods plant^-1^ and seed yield plant^-1^. In plant height, all 96 accessions were grouped into different classes (Fig 1) and only two accessions ILWL15 (63.50 cm) and ILWL19 (66.50 cm) of *L*. *nigricans* were reported for maximum height. Likewise, number of branches plant^-1^ was highest in accession ILWL 418 (52 branches) of *L*. *ervoides* and ILWL 480 (37 branches) of *L*. *orientalis*. The number of pods plant^-1^ ranged from 3 to 1167 pods plant^-1^ and only two accessions ILWL 418 (1002 pods) of *L*. *ervoides* and ILWL 18 (1167 pods) of *L*. *nigricans* revealed maximum number. The character seed yield plant^-1^ also showed variation and only two accessions ILWL 18 (13.60 g) and ILWL 19 (12.55 g) of *L*. *nigricans* showed maximum seed yield palnt^-1^.

### Diversity analysis

**Fig 2:**
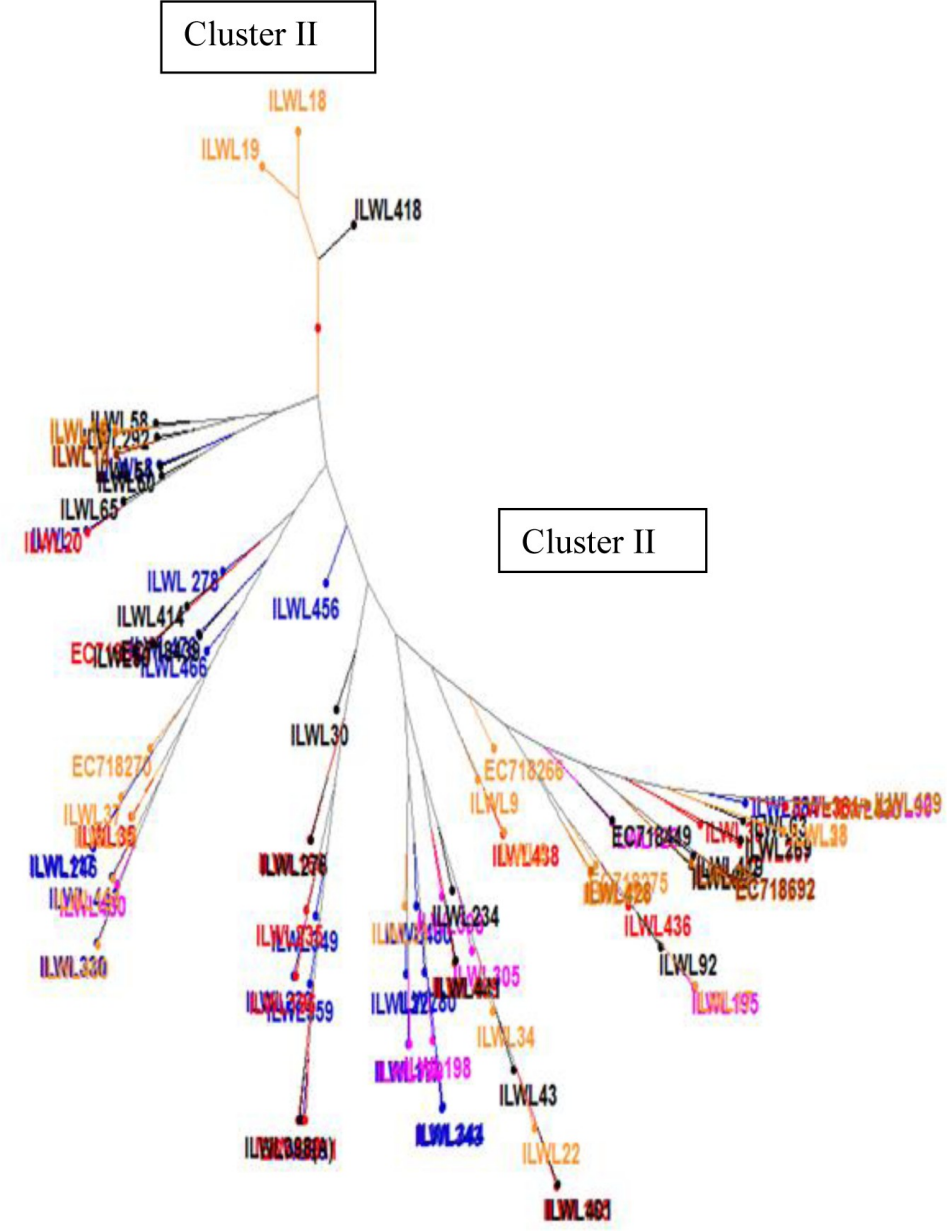
Hierarchical clustering of wild lentil accessions based on quantitative data analysis.

Wild lentil accessions formed two major clusters I and II in hierarchical clustering as shown in Fig 2. Major cluster I grouped only three accessions including ILWL418 (*L*. *ervoides*) from Syria; ILWL18 and ILWL19 (*L*. *nigricans*) from France and Spain, respectively. Cluster II contains all remaining accessions was further divided into A and B clusters. Bifurcation of Cluster A resulted into two sub clusters AI and AII, where sub cluster AI was occupied by *L*. *ervoides* accessions ILWL58 and ILWL292 from Turkey; *L*. *nigricans* accessions ILWL16 from Alpes Cote d’ Azur and ILWL15 from France; and ILWL14 and ILWL15 of *L*. *lamottei* from France. Sub cluster AII grouped ILWL7 and ILWL8 of *L*. *orientalis* from Turkey; ILWL51, ILWL60 and ILWL65 of *L*. *ervoides* including first from Montenegro and other two from Turkey; and ILWL20 of *L*. *odemensis* from Palestine. Cluster B was occupied by two sub clusters BI and BII, each of which were further divided into two groups a, b and c, d, respectively. Group d further formed outgroups (d1 and d2) and finally clutches (d2a and d2b). As Cluster II grouped sub-clusters were occupied by accessions from different wild lentil species belonging to different countries of origin, they showed highest diversity (S1 Table). Euclidean dissimilarity matrices varied from 6.01 between ILWL429 (*L*. *lamottei*) from Spain and ILWL90 (*L*. *tomentosus*) from Turkey to 2156.52 between ILWL18 (*L*. *nigricans*) from France and ILWL476 (*L*. *orientalis*) from Turkey (S1 Table). First two principal coordinates in factorial analysis explained 99.07% and 0.52% variability, respectively. In both type of groupings, the role of geographical or country of origin of accessions was not observed.

### Correlations among different parameters

The correlation coefficient among different agro-morphological traits of wild lentil accessions is shown in Table 2. Correlation indices highlighted a number of significant inter-relationships (both +ve and -ve) among studied traits. In the present study, significant positive correlation was observed between days to flowering and maturity (>0.4 r-value). Similarly, significant positive correlations were obtained between seed yield plant^-1^, biological yield plant^-1^ and harvest index. These traits also showed positive correlations with plant height (r^2^ <4.0 in case of biological yield plant^-1^), number of branches plant^-1^ (r^2^ <4.0 in case of harvest index), number of pods plant^-1^ and seeds plant^-1^. Seeds plant^-1^ showed significant correlation with plant height, branches plant^-1^ and pods plant^-1^. The number of pods plant^-1^ exhibited significant positive correlation with branches plant^-1^. Significant negative correlation was observed between days to flowering and seed yield plant^-1^, seeds plant^-1^, pods plant^-1^, and harvest index.

**Table 2:** Correlations among various agro-morphological traits of wild lentil accessions.

Traits	DF	DM	PH	NBPP	NPPP	NSPPL	NSPPD	SDWT	SYPP	BYPP	HI	RT	PM	FW
**DF**	1.00	.750(**)	-0.13	-.219(*)	-.542(**)	-.534(**)	-0.16	-0.06	-.495(**)	-.311(**)	-.543(**)	-0.15	-0.06	-0.15
**DM**	.750(**)	1.00	0.04	-0.15	-.346(**)	-.319(**)	-.256(*)	-0.09	-.350(**)	-.235(*)	-.319(**)	-0.02	0.03	-0.17
**PH**	-0.13	0.04	1.00	.242(*)	.360(**)	.411(**)	-0.11	-0.15	.468(**)	.344(**)	.438(**)	.342(**)	0.15	-0.14
**NBPP**	-.219(*)	-0.15	.242(*)	1.00	.544(**)	.536(**)	-.285(**)	-0.09	.504(**)	.539(**)	.388(**)	.328(**)	.272(**)	0.08
**NPPP**	-.542(**)	-.346(**)	.360(**)	.544(**)	1.00	.987(**)	-0.15	-0.09	.928(**)	.833(**)	.738(**)	.367(**)	.290(**)	0.03
**NSPPL**	-.534(**)	-.319(**)	.411(**)	.536(**)	.987(**)	1.00	-0.18	-0.08	.956(**)	.820(**)	.788(**)	.308(**)	.322(**)	-0.02
**NSPPD**	-0.16	-.256(*)	-0.11	-.285(**)	-0.15	-0.18	1.00	-0.12	-.244(*)	-.247(*)	-0.16	-.291(**)	-.224(*)	0.13
**SDWT**	-0.06	-0.09	-0.15	-0.09	-0.09	-0.08	-0.12	1.00	0.00	-0.12	0.02	-0.02	0.07	0.14
**SYPP**	-.495(**)	-.350(**)	.468(**)	.504(**)	.928(**)	.956(**)	-.244(*)	0.00	1.00	.814(**)	.841(**)	.373(**)	.391(**)	0.00
**BYPP**	-.311(**)	-.235(*)	.344(**)	.539(**)	.833(**)	.820(**)	-.247(*)	-0.12	.814(**)	1.00	.508(**)	.377(**)	.283(**)	-0.02
**HI**	-.543(**)	-.319(**)	.438(**)	.388(**)	.738(**)	.788(**)	-0.16	0.02	.841(**)	.508(**)	1.00	.312(**)	.347(**)	0.00
**Rust**	-0.15	-0.02	.342(**)	.328(**)	.367(**)	.308(**)	-.291(**)	-0.02	.373(**)	.377(**)	.312(**)	1.00	.314(**)	-0.08
**PM**	-0.06	0.03	0.15	.272(**)	.290(**)	.322(**)	-.224(*)	0.07	.391(**)	.283(**)	.347(**)	.314(**)	1.00	-0.02
**FW**	-0.15	-0.17	-0.14	0.08	0.03	-0.02	0.13	0.14	0.00	-0.02	0.00	-0.08	-0.02	1.00

*Correlation is significant at the 0.05 level

**Correlation is significant at the 0.01 level

DF, Days to flowering; DM, Days to maturity; PH, Plant height; NBPP, Number of branches plant^-1^; NPPP, Number of pods plant^-1^; NSPPL, Number of seeds plant^-1^; NSPPD, Number of seeds pod^-1^; SDWT, Seed weight; SYPP, Seed yield plant; BYPP, Biological yield plant; HI, Harvest index; RT, Rust; PM, Powdery mildew; FW, Fusarium wilt

### Principle component analysis

**Fig 3:**
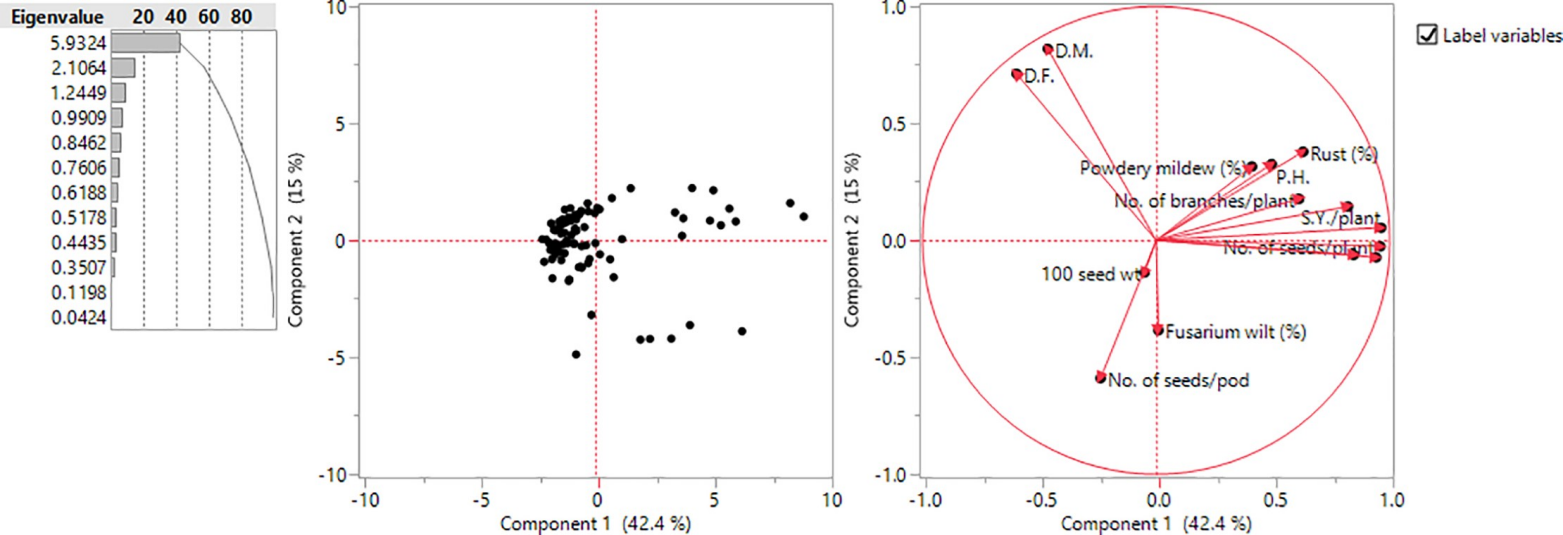
PCA analysis—(a) 2-D graph of first two principal components and (b) various traits contributing to the variability.

Maximum variability (66.31%) was explained by only three components with Eigenvalue >1.0 (Table 3). Of the total variation, 42.37% was exhibited by PC1, and the contributing traits with high coefficients included seed yield plant^-1^, seeds plant^-1^, pods plant^-1^, harvest index and biological yield plant^-1^. Similarly, 15.04% variability was explained by PC2, and the contributing traits with high coefficients included days to maturity, days to flowering, rust incidence and plant height. Similarly, PC3 contributed 8.89% of overall variability, and the traits contributing to diversity included 100- seed weight, Fusarium wilt and Powdery mildew severity. Fig 3A and 3B shows the two dimensional variability pattern and contributing traits towards variability, respectively.

**Table 3:** Eigen vectors, eigenvalues, individual and cumulative percentages of variation explained by the first three principal components (PC) of wild lentil accessions.

Traits	Prin1	Prin2	Prin3
Days to flowering	-0.25	0.49	0.00
Days to maturity	-0.19	0.56	-0.01
Plant height (cm)	0.20	0.22	-0.30
Number of branches plant^-1^	0.25	0.12	0.09
Number of pods plant^-1^	0.39	-0.05	-0.06
Number of seeds plant^-1^	0.39	-0.02	-0.06
Number of seeds pod^-1^	-0.10	-0.41	-0.37
100 -seed weight (g)	-0.02	-0.09	0.71
Seed yield plant^-1^ (g)	0.40	0.04	0.02
Biological yield plant^-1^ (g)	0.34	0.10	-0.06
Harvest index (%)	0.35	-0.04	0.02
Rust severity	0.26	0.26	0.04
Powdery mildew severity	0.17	0.22	0.33
Fusarium wilt	0.00	-0.26	0.37
Eigen value	5.93	2.11	1.24
Percent	42.37	15.04	8.89
Cum. Percentage	42.37	57.42	66.31

### Resistance to key biotic stresses

#### Rust resistance

**Fig 4:**
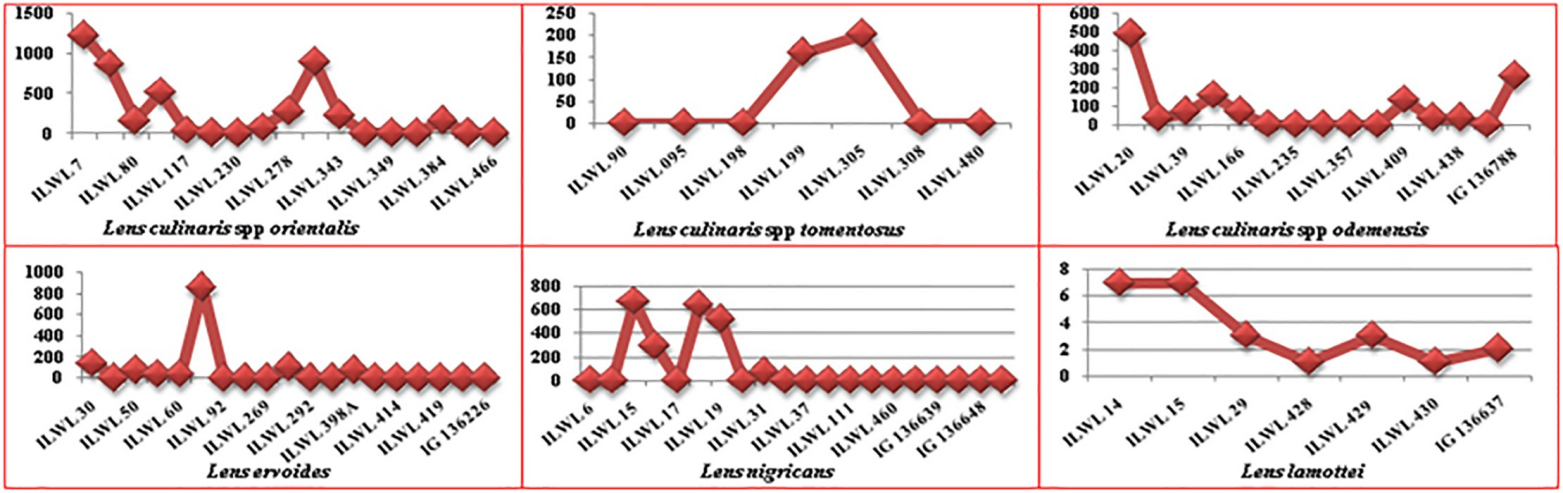
Screening of wild lentil accessions against rust resistance.

Majority of wild lentil accessions were resistant and moderately resistant against rust under controlled artificial inoculation conditions. Accessions ILWL90, ILWL195, ILWL198 and ILWL480 (*L*. *tomentosus*); ILWL230, 349, 476 (*L*. *orientalis*); ILWL203, 235 and 357 (*L*. *odemensis*); ILWL60, 204, 292, 414 and 442 (*L*. *ervoides*); and ILWL16, 18, 37, 460 and EC718266 (*L*. *nigricans*) were identified as resistant (Tables 4 and 5). The remaining accessions were scored as moderately resistant, susceptible and some of them were highly susceptible against the pathogen. The distribution of accessions into various reaction categories based on disease score is presented in Fig 4.

**Table 4:** Range of variation of wild lentil accessions against rust, powdery mildew and *Fusarium* wilt.

Species	Rust			Powdery mildew			Fusarium wilt		
	Range	Mean ±SE	CV (%)	Range	Mean ±SE	CV (%)	Range	Mean ±SE	CV (%)
***L*. *culinaris***	1–1	1.00±0.00	0.00	0–5	5.00±0.00	0.00	1–7	4.00±3.00	106.07
***L*. *orientalis***	1–9	3.41±0.70	84.23	0–5	3.56±0.35	42.29	1–9	4.44±0.74	70.40
***L*. *tomentosus***	1–5	1.86±0.59	84.73	0–5	2.29±0.75	86.45	1–9	5.00±1.15	61.10
***L*. *odemensis***	1–6	2.33±0.40	66.13	0–4	1.60±0.39	93.90	1–9	5.67±0.64	43.57
***L*. *ervoides***	1–8	1.95±0.42	92.93	0–5	1.90±0.45	105.12	1–9	5.18±0.72	65.14
***L*. *nigricans***	1–7	2.10±0.46	98.81	0–5	2.52±0.52	95.11	1–9	4.43±0.69	71.61
***L*. *lamottei***	1–7	3.43±0.97	75.04	0–5	3.29±0.75	60.14	1–9	4.43±1.04	62.33

**Table 5:** Identification of donor accessions for their introgression to lentil genetic improvement against important agronomic and major biotic stress related traits.

Taxa/ trait	Accession	Gene pool	Country origin
Agronomic			
*L*. *orientalis*	ILWL255, 476 ((High pods plant^-1^, shorter internode)	Primary	Turkey, Syria
*L*. *odemensis*	ILWL203, 237 (High pods plant^-1^, shorter internode)	Primary	Syria
*L*. *ervoides*	ILWL294, 321 ((High pods plant^-1^, shorter internode)	Tertiary	Turkey, Syria
**Rust**			
*L*. *orientalis*	ILWL230, 349, 466	Primary	Turkey
*L*. *odemensis*	ILWL235, 357, ILWL203	Primary	Syria
*L*. *tomentosus*	ILWL 90, 95,198, 308, 480	Primary	Syria
*L*. *ervoides*	ILWL60, 92, 269, 292, 414, 419, IG136226	Tertiary	Turkey, Syria
*L*. *nigricans*	ILWL9, 17, 37, 111, 460, IG136639, IG136648	Quaternary	
**Powdery mildew**			
*L*. *orientalis*	ILWL 230, 466	Primary	Syria
*L*. *odemensis*	ILWL 39, 196, IG136788	Primary	Turkey, Syria
*L*. *tomentosus*	ILWL 198, 480	Primary	Turkey, Syria
*L*. *ervoides*	ILWL51, 401, 418, 441	Tertiary	Montenegro, Lebanon, Syria, Turkey
*L*. *nigricans*	ILWL6, 9, 22, 34, 37, 111	Quaternary	Turkey, Syria Italy, Ukraine, Turkey, Turkey
*L*. *lamottei*	ILWL 29	Secondary	Spain
**Fusarium wilt**			
*L*. *orientalis*	ILWL7, 96, 117, 227, 246, 344, 359	Primary	Turkey, Turkey, Syria, Syria, Syria, Syria, Syria,
*L*. *odemensis*	ILWL15, 39	Primary	France, Turkey
*L*. *tomentosus*	ILWL 199, 308	Primary	
*L*. *ervoides*	ILWL 58, 398, 414, IG136626	Tertiary	Turkey, Lebanon, Syria Israeli
*L*. *nigricans*	ILWL17, 19, 23, 34, 38, 474	Quaternary	Spain, Italy, Ukraine, Turkey, Turkey
*L*. *lamottei*	ILWL 20, 430	Secondary	PAL, Spain,

Gene pool classification of Wong et al. [31]

#### Powdery mildew resistance

**Fig 5:**
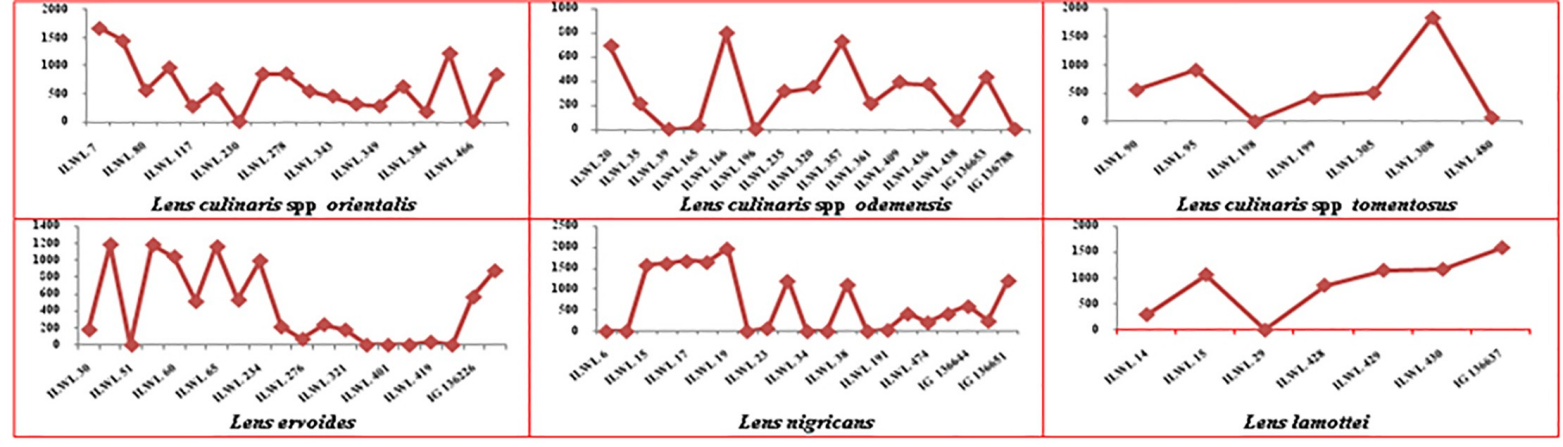
Screening of wild lentil accessions against powdery mildew resistance.

Out of 24 accessions of *L*. *orientalis*, ILWL 230 and ILWL 476 were found highly resistant. Likewise, in *L*. *odemensis*, accessions ILWL 39, ILWL 203, and IG 136788; ILWL 198 and ILWL 480 of *L*. *tomentosus* were also found to be highly resistant (Tables 4 and 5). However, accessions ILWL 51, ILWL 401, ILWL 418 and ILWL 441 of *L*. *ervoides*; ILWL9, ILWL22, ILWL34, ILWL37 and ILWL191 of *L*. *nigricans*; and ILWL 29 of *L*. *lamottei* with disease reaction 2 were found to be resistant. The remaining accessions were either moderately resistant or susceptible against the pathogen (Fig 5).

#### Fusarium wilt resistance

**Fig 6:**
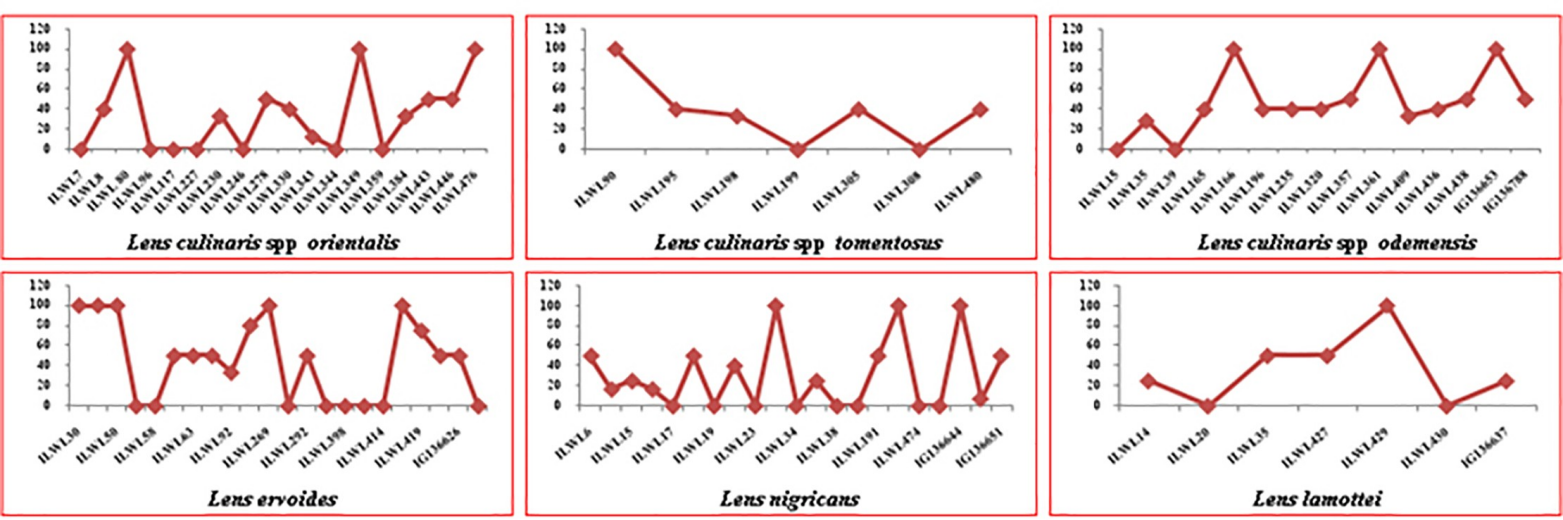
Screening of wild lentil accessions against Fusarium wilt resistance.

The wild lentil accessions, which showed resistant disease reaction to Fusarium wilt included ILWL7, ILWL96, ILWL117, ILWL227, ILWL246, ILWL344 and ILWL359 (*L*. *orientalis*); ILWL15 and ILWL39 (*L*. *odemensis*); ILWL199 and ILWL 308 (*L*. *tomentosus*). Accessions ILWL58, ILWL398, ILWL414, IG136626 (*L*. *ervoides*); ILWL17, ILWL19, ILWL23, ILWL34, ILWL38 and ILWL474 (*L*. *nigricans*) and ILWL20 and ILWL430 (*L*. *lamottei*) were found resistant against the wilt (Tables 4 and 5). Some accessions were moderately resistant and the remaining was susceptible against the pathogen as depicted in Fig 6.

## Discussion

The precise evaluation of crop wild relatives is pre-requisite to identify target traits of interest followed by their introgression into the background of cultivated varieties for enhancing genetic gains [20–22]. A wide range of variation in wild lentil accessions was observed against the target traits viz. earliness, high pod number and seed yield suggesting diverse genetic makeup and geographical origins of wild lentil collections. These promising accessions belonging to different taxa and ecological niches could be useful resource for enhancing genetic gains of cultivated varieties. Accessions ILWL 18 and ILWL 19 of *L*. *nigricans* were found promising for high seed yield plant^-1^, suggesting their broader genetic base as also reflected in clustering pattern of wild accessions based on quantitative data analysis. Euclidean dissimilarity matrices of wild lentil accessions ranged from 6.01 to 2156.52, which revealed very high variability among interspecific lentil taxa. The observed variability could be due to the presence of diverse origins of these wild accessions. Among the different countries of origin, accessions from Turkey showed maximum variability for the studied traits suggesting more exploration and collection of lentil germplasm. Viera et al. [23] characterized wheat germplasm based on phenotypic traits and obtained distances up to 196.61. The report of existing variability among germplasm accessions provides an idea about the expected heterosis for starting breeding programme. A broad range of Euclidean distances obtained in the present study shows the importance of wild lentil accessions as a source of diverse heterotic material [24]. However, the diversity pattern further suggested that no role was played by the geographical distributions of global accessions in their grouping. This grouping pattern can be described on the basis of different genetic constitution of accessions. Further, it might be due to continuous gene flow occurrence among wild lentil taxa [8]. No role of geographical distributions was observed in grouping pattern of accessions clustered on the basis of morphological, biochemical and molecular markers in guar [25]. Similar observation in wild lentil accessions was also reported by Singh et al. [8] and Kumar et al. [26]. Further, significant positive correlation of seed yield plant^-1^ with seeds plant^-1^, pods plant^-1^, branches plant^-1^ was observed in lentil accessions and similar correlations have also been reported by other workers [27–29]. They reported that seeds plant^-1^, biological yield plant^-1^, pods plant^-1^, and harvest index are the important yield component traits in lentil contributing towards yield enhancement. Such correlations are useful for lentil genetic improvement and breeding programmes. These observations were found to be in accordance with the results of Toklu et al. [29] and Abo-Hegazy et al. [30]. The traits which contributed heavily towards variability included seed yield plant^-1^, seeds pod^-1^, pods plant^-1^, biological yield plant^-1^ and harvest index. These findings highlighted the significance of yield related component characters generating substantial variations in wild lentil accessions.

Furthermore, wild taxa of lentil are known to be resistant against major biotic and abiotic stresses [8]. They are the carrier of important characters and have potential value in diversification of cultivated gene pool for enhancing genetic gains. Some wild lentil accessions revealed resistant disease reaction against more than one disease (Table 5) viz. accessions ILWL230 and ILWL476 of *L*. *orientalis* (rust and powdery mildew); accessions ILWL9 and ILWL37 of *L*. *nigricans* (rust and powdery mildew); accession IG136639 of *L*. *ervoides* (powdery mildew and Fusarium wilt) and accession ILWL308 of *L*. *tomentosus* (rust and Fusarium wilt). It is important to mention that the northwestern part has been the traditional lentil cultivation area in India. But in late 1980s, among various factors including the epidemics of rust, powdery mildew and Fusarium wilt resulted in drastic reduction of lentil area and production as well. In this context, gene sources identified against rust, powdery mildew and *Fusarium* wilt in the present study can prove to be useful donors which could be exploited through pre-breeding with the aim to develop usable germplasm for developing resistant lentil varieties, especially for the epidemiologically important Indian conditions. Furthermore, the development of lentil cultivars with combined resistance against major biotic stresses may help in the development of appropriate management strategies in future in conjunction with allelism studies to identify and pyramid novel genes for resistance breeding of lentil.

## Conclusions

During the past few years, little efforts were made on evaluation of wild lentil genetic resources in India. However, this is the first attempt on characterization and evaluation of all available global wild lentil germplasm against the target traits of interest under diverse agro-ecological conditions. Further, the study has helped in identifying the confirmed and stable gene sources (donors) across intra and interspecific accessions viz. ILWL203 (EC789113) of *L*. *odemensis* for rust and high pod number; ILWL230 (EC718515), ILWL476 (EC718605) of *L*. *orientalis* for rust and powdery mildew; ILWL191 (EC718682), ILWL9 (EC718236), and ILWL37 (EC718257) of *L*. *nigricans* for rust and powdery mildew, IG136639 of *L*. *ervoides* for powdery mildew and Fusarium wilt and ILWL308 (EC728782) of *L*. *tomentosus* for rust and Fusarium wilt. These gene sources could be taken in lentil wide hybridization programme for enhancing genetic gains of cultivated varieties and could also be shared among lentil breeders/researchers in the country or elsewhere under Standard Material Transfer Agreement (SMTA) for strengthening the on-going lentil genetic improvement programmes.

## Supporting information

S1 Table Clustering pattern of wild lentil core accessions based on agro-morphological characters. (DOCX)

## Abbreviations

NBPGR: National Bureau of Plant Genetic Resources
NARS: National Agricultural Research System
ICARDA: International Centre for Agricultural Research in Dry Areas
PM: Powdery Mildew
FW: Fusarium Wilt
ILWL: International Legume Wild Lentil
PCA: Principle Component Analysis
PC: Principle Component
BIGM: Biodiversity and Integrated Gene Management
CWRs: Crop Wild Relatives
RCBD: Randomized Complete Block Design
UPGMA: Unpaired Group Method Analysis
SMTA: Standard Material Transfer Agreement

## References

[1] Arumuganathan K., Earle ED. Nuclear DNA content of some important plant species. Plant Mol. Biol Rep.1991; 9: 208–218.

[2] Ferguson ME, Maxted N, Slageren MV, Robertson LD. A re-assessment of the taxonomy of Lens. Bot. J. Linn. Soc. 2000; 133: 41–59.

[3] Food and Agriculture Organization (FAO) FAO Statistical database of the United Nations, Food and Agriculture Organization Rome; 2017.

[4] Erskine W, Muehlbauer FJ, Sarker A, Sharma B. An Introduction, In: Erskine W., Muehlbauer F.J., Sarker A. and Sharma B.: The lentil, botany, production and uses. CAB International; 2009 p.1–3.

[5] Kumar S, Gupta S, Chandra S, Singh BB. How wide is the genetic base of pulse crops, Pulses in New Perspective In: Ali M, Singh BB, Kumar S, Dhar V eds. Indian Society of Pulses Research and Development, Indian Institute of Pulses Research, Kanpur India; 2003.

[6] Maxted N, Ford BV, Kell SP, Iriondo JM, Dulloo ME. Crop wild relative conservation and use, Wallingford, UK, CABI publishing; 2008. 10.1186/1471-2105-9-116 PMC2375123 18298814

[7] Lane A, Jarvis A. Changes in climate will modify the geography of crop suitability: Agricultural biodiversity can help with adaptation. Climate Proofing Innovation for Poverty Reduction and Food Security, ICRISAT, Patancheru; 2007.

[8] Singh M, Bisht IS, Kumar S, Dutta M, Bansal KC, Sarker A, et al Global wild annual Lens collection: a potential resource for lentil genetic base broadening and yield enhancement. PLoS ONE, (2014) 10.1371/journal.pone.0107781 25254552 PMC4177869

[9] SAS. Sas Institute Inc. Cary, NC, SAS Institute; 2011.

[10] Burton GW. Quantitative inheritance in grasses, in Proceedings of the 454 International Grassland Congress, State College, PA: Pennsylvania State College; 1952.

[11] Lush JL. Intrusive collection of regression of offspring on dams as a method 501 of estimating heritability of characters. Proc. Am. Soc. Anim. Prod. 1940; 33: 293–301.

[12] Johnson HW, Robinson HF, Comstock RE. Estimates of genetic and environmental variability in Soybean. Agron. 1955; J. 47: 314–318.

[13] Perrier X, Flori A, Bonnot F. Data analysis methods, In: Hamon P., Seguin M., Perrier X., Glaszmann J.C. Ed., Genetic diversity of cultivated tropical plants. Enfield, Science Publishers Montpellier; 2003.

[14] Mayee CD, Datar VV. Phytopathometry Technical Bulletin-1, Marathwada Agricultural University, Parbhani; 1986.

[15] Tiwari KR, Penner GA, Warkentin TD, Rashid KY. Pathogenic variation in Erisiphe polygoni, the casual organism of powdery mildew of lentil. Can. J. Plant Pathol. 1997; 19: 267–271.

[16] Bayaa B, Erskine W. A screening technique for resistance to vascular wilt in lentil. Arab J. Plant Prot. 1990; 8: 30–33.

[17] Bayaa B, Erskine W, Hamdi A. Evaluation of wilt lentil collection for resistance to vascular wilt. Genet. Resour. Crop Evol. 1995; 42: 231–235.

[18] Bayaa B, Erskine W, Singh M. Screening lentil for resistance to Fusarium wilt: methodology and sources of resistance. Euphytica. 1997; 98: 69–74.

[19] Eujayl I, Erskine W, Bayaa B, Baum M, Pehu E. Fusarium vascular wilt in lentil: inheritance and identification of DNA markers for resistance. Plant Breed. 1998; 117: 497–499.

[20] Duvick DN. Genetic diversity in major farm crops on the farm and in reverse. Eco. Bot. 1984; 38: 151–178.

[21] Lazaro A, Ruiz M, Rosa L, Martin I. Relationship between agro-morphological characters and climate parameters in Spanish landraces of lentil. Genet Resour Crop Evol. 2001; 48: 239–249.

[22] Naghavi MR, Jahansouz MR. Variation in the agronomic and morphological traits of lentil accessions. J. Integ. Plant Biol. 2005; 47: 375–379.

[23] Vieira EA, Carvalho FIF, Bertan de I, Kopp M, Zimmer PD. Association between genetic distances in wheat as estimated by AFLP and morphological markers. Genet. Mol. Biol. 2007; 30: 392–399.

[24] Gupta SK, Nepolean T, Shaikh CG, Rai K, Hash CT, Das RR, et al Phenotypic and molecular diversity-based prediction of heterosis in pearl millet (Pennisetum glaucum L. (R.) Br.).The Crop Journal. 2018; 6: 271–281.

[25] Kumar S, Joshi UN, Singh V, Singh JV, Saini ML. Characterization of released and elite genotypes of guar from Indian proves unrelated to geographical origin. Genet Resour Crop Evol. 2013; 60: 2017–2032.

[26] Kumar S, Choudhary AK, Rana KS Sarker A, Singh M. Biofortification potential of global wild annual lentil core collection. PLOS ONE. 2018; 10.1371/journal.pone.0191122. PMC5773171 29346404

[27] Luthra SK, Sharma PC. Correlation and path analysis in lentil. Lens New Letter. 1990; 7: 5–8.

[28] Sarwar G, Abbas G, Asghar MJ. Quantitative analysis of yield related traits in lentil. J. Agric. Res. 2013; 51: 239–246.

[29] Toklu F, Ozkan H, Karakoy T, Coyne C. Evaluation of advanced lentil lines for diversity in seed mineral concentration, grain yield and yield components. J Agric. Sci. 2015; 23: 213–222.

[30] Abo-Hegazy SRE, Selim T, El-Emam EAA. Correlation and path co-efficient analysis of yield and some yield components in lentil. Egyptian J. Pl. Breed. 2012; 16: 147–159.

[31] Wong MM, Gujaria N, Ramsay L, Yuan HY, Caron C, Diapari M, et al Classification and characterization of species within the genus *Lens* using genotyping-by- sequencing. PLoS ONE. 2015; 10(3) e0122025 10.1371/journal.pone.0122025 25815480 PMC4376907

