# Supplementary material for: Evaluation and identification of wild lentil accessions for enhancing genetic gains of cultivated varieties
Source: PLoS One. 2020 Mar 3;15(3):e0229554. doi: 10.1371/journal.pone.0229554 (PMC7053756; doi:10.1371/journal.pone.0229554)
Supplement: S1 Table — (DOCX) [file pone.0229554.s001.docx]

**Supplementary table 1: Clustering pattern of wild lentil core accessions based on agro-morphological characters**

| **Major cluster** | **Cluster** | **Sub cluster** | **groups** | **Outgroups** | **clutch** | **Accessions** | **Species** |
| --- | --- | --- | --- | --- | --- | --- | --- |
| I |  |  |  |  |  | ILWL418, ILWL18 and ILWL19 | *L. ervoides* and *L. nigricans* |
| II | A | AI |  |  |  | ILWL58, ILWL16, ILWL15, ILWL292, ILWL15 and ILWL14 | *L. ervoides, L. nigricans, L. lamottei* |
|  |  | AII |  |  |  | ILWL8, ILWL51, ILWL60, ILWL65, ILWL7 and ILWL20 | *L. culinaris* ssp*. orientalis,* *L. ervoides*,  *L. culinaris* ssp. *odemensis* |
|  | B | BI | a |  |  | ILWL278, ILWL414, EC718694 and ILWL50 | *L. culinaris* ssp. *orientalis, L. ervoides*,  *L. culinaris* ssp. *odemensis* |
|  |  |  | b |  |  | ILWL476, EC718439, ILWL466, EC718270, ILWL37, ILWL117, ILWL246, ILWL35, ILWL111, ILWL443, ILWL191, ILWL480, ILWL330 and ILWL460 | *L. culinaris* ssp. *orientalis, L. ervoides, L. nigricans, L. culinaris* ssp. *Odemensis*,  *L. culinaris* ssp. *tomentosus* |
|  |  | BII | c |  |  | ILWL456 | *L. culinaris* ssp. *orientalis* |
|  |  |  | d | d1 |  | ILWL30, ILWL166, ILWL276, ILWL235, ILWL230, ILWL196, ILWL349, ILWL359, ILWL320, ILWL398, ILWL96 and EC718311 | *L. ervoides, L. culinaris* ssp*. odemensis* ,  *L. culinaris* ssp. *orientalis* |
|  |  |  |  | d2 | d2a | ILWL31, ILWL227, ILWL117, ILWL199, ILWL480, ILWL80, ILWL198, ILWL343, ILWL344, ILWL308, ILWL409, ILWL441, ILWL234, ILWL305, ILWL34, ILWL43, ILWL22, ILWL165 and ILWL401 | *L. nigricans, L. culinaris* ssp. *orientalis, L. culinaris* ssp*. tomentosus, L. culinaris* ssp. *odemensis* and *L. ervoides* |
|  |  |  |  |  | d2b | ILWL9, ILWL438, ILWL6, EC718266, ILWL474, ILWL428, EC718275, ILWL436, ILWL92, ILWL195, ILWL17, ILWL120, EC718449, ILWL321, ILWL29, ILWL419, EC718273, EC718692, ILWL39, ILWL357, ILWL269, ILWL63, ILWL23, ILWL38, ILWL384, ILWL361, ILWL430, ILWL90 and ILWL429 | *L. nigricans, L. culinaris* ssp. *odemensis, L. lamottei, L. ervoides, L. culinaris* ssp. *tomentosus* , *L. culinaris* ssp*. orientalis* |
