# Supplementary material for: Evaluation and identification of wild lentil accessions for enhancing genetic gains of cultivated varieties
Source: PLoS One. 2020 Mar 3;15(3):e0229554. doi: 10.1371/journal.pone.0229554 (PMC7053756; doi:10.1371/journal.pone.0229554)
Supplement: Attachment — Submitted filename: PONE-D-19-35002_reviewer.doc [file pone.0229554.s003.doc]

**PLOS ONE**

**Evaluation and identification of wild lentil accessions for enhancing genetic gains of cultivated varieties**

|  |  |  | --Manuscript Draft-- |
| --- | --- | --- | --- |
|  |  |  |  |
| **Manuscript Number:** | | | PONE-D-19-35002 |
| **Article Type:** | | | Research Article |
|  |  |  |  |
| **Full Title:** | | | Evaluation and identification of wild lentil accessions for enhancing genetic gains of |
|  |  |  | cultivated varieties |
| **Short Title:** | | | Evaluation of wild Lens taxa |
|  |  |  |  |
| **Corresponding Author:** | | | Mohar Singh, Ph.D. Plant Breeding |
|  |  |  | National Bureau of Plant Genetic Resources, Pusa India |
|  |  |  | New Delhi, New Delhi INDIA |
| **Keywords:** | | | Wild lentil, germplasm enhancement, agronomic evaluation, biotic stresses, elite |
|  |  |  | donors |
|  |  |  |  |
| **Abstract:** | | | Domesticated lentil has a relatively narrow genetic base globally and most released |
|  |  |  | varieties are susceptible to severe biotic and abiotic stresses. The crop wild relatives |
|  |  |  | could provide new traits of interest for tailoring novel germplasm and cultivated lentil |
|  |  |  | improvement. The primary objective of this study was to evaluate wild lentil accessions |
|  |  |  | for identification of economically viable agro-morphological traits and resistance |
|  |  |  | against major biotic stresses. The study has revealed substantial variations in seed |
|  |  |  | yield and its important component characters. Further, the diversity analysis of wild |
|  |  |  | accessions showed two major clusters which were bifurcated into sub-clusters, thereby |
|  |  |  | suggesting their wider genetic divergence. However, principal component analysis |
|  |  |  | exhibited that seed yield plant -1 , number of seeds plant -1 , number of pods plant - |
|  |  |  | 1 , harvest index and biological yield plant -1 contributed significantly to the total |
|  |  |  | genetic variation assessed in wild lentil taxa. Moreover, some of the wild accessions |
|  |  |  | collected from Syria and Turkey regions showed resistance against more than one |
|  |  |  | disease indicating rich diversity of lentil genetic resources. The identification of most |
|  |  |  | promising genotypes carrying resistance against major biotic stresses could be utilized |
|  |  |  | in the cultivated or susceptible varieties of lentil for enhancing genetic gains. The study |
|  |  |  | has also identified some trait specific accessions, which could also be taken into the |
|  |  |  | consideration while planning distant hybridization in lentil. |
| **Order of Authors:** | | | Mohar Singh, Ph.D. Plant Breeding |
|  |  |  |  |
|  |  |  | Sandeep Kumar |
|  |  |  |  |
|  |  |  | Ashwani Kumar Basandrai |
|  |  |  |  |
|  |  |  | Daisy Basandrai |
|  |  |  |  |
|  |  |  | Nikhil Malhotra |
|  |  |  |  |
|  |  |  | Deep Rattan Saxena |
|  |  |  |  |
|  |  |  | Dorin Gupta |
|  |  |  |  |
|  |  |  | Ashutosh Sarker |
|  |  |  |  |
|  |  |  | Kuldeep Singh |
|  |  |  |  |
| **Additional Information:** | | |  |
| **Question** | | | **Response** |
|  |  |  |  |
| **Financial Disclosure** | | | AS; Ashutosh Sarker, ICARDA office will make publication charges available with him |
| Enter a financial disclosure statement that | | |  |
| describes the sources of funding for the | | |  |
| work included in this submission. Review | | |  |
| the [submission guidelines](http://journals.plos.org/plosone/s/submission-guidelines#loc-financial-disclosure-statement) for detailed | | |  |
|  |  |  |  |

*Powered by Editorial Manager® and ProduXion Manager® from Aries Systems Corporation*

requirements. View published research

articles from [*PLOS ONE*](http://journals.plos.org/plosone/) for specific

examples.

This statement is required for submission

and **will appear in the published article** if

the submission is accepted. Please make

sure it is accurate.

**Unfunded studies**

Enter: *The author(s) received no specific*

*funding for this work.*

**Funded studies**

Enter a statement with the following details:

- Initials of the authors who received each award
- Grant numbers awarded to each author
- The full name of each funder
- URL of each funder website
- Did the sponsors or funders play any role in the study design, data collection and analysis, decision to publish, or preparation of the manuscript?
- **NO** - Include this sentence at the end of your statement: *The funders had no role in* *study design, data collection and analysis, decision to publish, or preparation of the manuscript.*
- **YES** - Specify the role(s) played.
- typeset

| **Competing Interests** | authors have no competing interests |
| --- | --- |

Use the instructions below to enter a

competing interest statement for this

submission. On behalf of all authors,

disclose any [competing interests](http://journals.plos.org/plosone/s/competing-interests) that

could be perceived to bias this

work—acknowledging all financial support

and any other relevant financial or non-

financial competing interests.

This statement **will appear in the**

**published article** if the submission is

accepted. Please make sure it is

accurate. View published research articles

from [*PLOS ONE*](http://journals.plos.org/plosone/) for specific examples.

*Powered by Editorial Manager® and ProduXion Manager® from Aries Systems Corporation*

**NO authors have competing interests**

Enter: *The authors have declared that no*

*competing interests exist.*

**Authors with competing interests**

Enter competing interest details beginning

with this statement:

*I have read the journal's policy and the*

*authors of this manuscript have the following*

*competing interests: [insert competing*

*interests here]*

* typeset

| **Ethics Statement** | The genetic materials was meant for research purpose and no need any permission |
| --- | --- |

Enter an ethics statement for this

submission. This statement is required if

the study involved:

- Human participants
- Human specimens or tissue
- Vertebrate animals or cephalopods
- Vertebrate embryos or tissues
- Field research

Write "N/A" if the submission does not

require an ethics statement.

General guidance is provided below.

Consult the [submission guidelines](http://journals.plos.org/plosone/s/submission-guidelines#loc-guidelines-for-specific-study-types) for

detailed instructions. **Make sure that all**

**information entered here is included in the**

**Methods section of the manuscript.**

*Powered by Editorial Manager® and ProduXion Manager® from Aries Systems Corporation*

**Format for specific study types**

**Human Subject Research (involving human**

**participants and/or tissue)**

- Give the name of the institutional review board or ethics committee that approved the study
- Include the approval number and/or a statement indicating approval of this research
- Indicate the form of consent obtained (written/oral) or the reason that consent was not obtained (e.g. the data were analyzed anonymously)

**Animal Research (involving vertebrate**

**animals, embryos or tissues)**

- Provide the name of the Institutional Animal Care and Use Committee (IACUC) or other relevant ethics board that reviewed the study protocol, and indicate whether they approved this research or granted a formal waiver of ethical approval
- Include an approval number if one was obtained
- If the study involved *non-human primates*, add *additional details* about animal welfare and steps taken to ameliorate suffering
- If anesthesia, euthanasia, or any kind of animal sacrifice is part of the study, include briefly which substances and/or methods were applied

**Field Research**

Include the following details if this study

involves the collection of plant, animal, or

other materials from a natural setting:

- Field permit number
- Name of the institution or relevant body that granted permission

| **Data Availability** | Yes - all data are fully available without restriction |
| --- | --- |

Authors are required to make all data

underlying the findings described fully

available, without restriction, and from the

time of publication. PLOS allows rare

exceptions to address legal and ethical

concerns. See the [PLOS Data Policy](http://journals.plos.org/plosone/s/data-availability) and

[FAQ](http://journals.plos.org/plosone/s/data-availability#loc-faqs-for-data-policy) for detailed information.

*Powered by Editorial Manager® and ProduXion Manager® from Aries Systems Corporation*

A Data Availability Statement describing

where the data can be found is required at

submission. Your answers to this question

constitute the Data Availability Statement

and **will be published in the article**, if

accepted.

**Important:** Stating ‘data available on request

from the author’ is not sufficient. If your data

are only available upon request, select ‘No’ for

the first question and explain your exceptional

situation in the text box.

Do the authors confirm that all data

underlying the findings described in their

manuscript are fully available without

restriction?

**Describe where the data may be found in**	as per norms

**full sentences. If you are copying our**

**sample text, replace any instances of XXX**

**with the appropriate details.**

- If the data are **held or will be held in a** **public repository**, include URLs, accession numbers or DOIs. If this information will only be available after acceptance, indicate this by ticking the box below. For example: *All* *XXX* *files* *are available from the XXX database (accession number(s) XXX, XXX.*).
- If the data are all contained **within the** **manuscript and/or Supporting Information files**, enter the following: *All relevant data are within the manuscript and its Supporting Information files.*
- If neither of these applies but you are able to provide **details of access** **elsewhere**, with or without limitations, please do so. For example:

*Data cannot be shared publicly because of [XXX]. Data are available from the XXX Institutional Data Access / Ethics Committee (contact via XXX) for researchers who meet the criteria for access to confidential data.*

*The data underlying the results*

*presented in the study are available*

*from (include the name of the third party*

Powered by Editorial Manager® and ProduXion Manager® from Aries Systems Corporation

| *and contact information or URL).* |  |
| --- | --- |
| • This text is appropriate if the data are |  |
| owned by a third party and authors do |  |
| not have permission to share the data. |  |
| * typeset |  |
| Additional data availability information: | Tick here if the URLs/accession numbers/DOIs will be available only after acceptance |
|  | of the manuscript for publication so that we can ensure their inclusion before |
|  | publication. |
|  |  |

*Powered by Editorial Manager® and ProduXion Manager® from Aries Systems Corporation*

Manuscript

[Click here to access/download;Manuscript;MS PLoS One-revised-clean.doc](https://www.editorialmanager.com/pone/download.aspx?id=25794309&guid=dea45645-7c6a-4c6d-b400-635ecd5aafb6&scheme=1)

**Evaluation and identification of wild lentil accessions for enhancing genetic gains of cultivated varieties**

Mohar Singh^1^*, Sandeep Kumar^2^, Ashwani Kumar Basandrai^3^, Daisy Basandrai^3^, Nikhil Malhotra^1^, Deep Rattan Saxena^4^, Dorin Gupta^5^, Ashutosh Sarker^6^ and Kuldeep Singh^2^

1. National Bureau of Plant Genetic Resources Regional Station, Shimla-171 004, India
2. National Bureau of Plant Genetic Resources, Pusa New Delhi, 110 012, India
3. CSK Himachal Pradesh Agricultural University Palampur-176 062, India
4. Rafi Ahmad Kidwai, College of Agriculture Sehore, India
5. Faculty of Veterinary and Agricultural Sciences, The University of Melbourne, Melbourne, Australia
6. South Asia and China Regional Programme of International Centre for Agricultural Research in Dry Area, DPS Marg, Pusa New Delhi-110 012, India

*Corresponding author

Dr Mohar Singh

ICAR-NBPGR Regional Station Shimla- 171 004, India

**Abstract**

Domesticated lentil has a relatively narrow genetic base globally and most released varieties are susceptible to severe biotic and abiotic stresses. The crop wild relatives could provide new traits of interest for tailoring novel germplasm and cultivated lentil improvement. The primary objective of this study was to evaluate wild lentil accessions for identification of economically viable agro-morphological traits and resistance against major biotic stresses. The study has revealed substantial variations in seed yield and its important component characters. Further, the diversity analysis of wild accessions showed two major clusters which were bifurcated into sub-clusters, thereby suggesting their wider genetic divergence. However, principal component analysis exhibited that seed yield plant^-1^, number of seeds plant^-1^, number of pods plant^-1^, harvest index and biological yield plant^-1^ contributed significantly to the total genetic variation assessed in wild lentil taxa. Moreover, some of the wild accessions collected from Syria and Turkey regions showed resistance against more than one disease indicating rich diversity of lentil genetic resources. The identification of most promising genotypes carrying resistance against major biotic stresses could be utilized in the cultivated or susceptible varieties of lentil for enhancing genetic gains. The study has also identified some trait specific accessions, which could also be taken into the consideration while planning distant hybridization in lentil.

**Key words**: wild lentil, germplasm enhancement, agronomic evaluation, biotic stresses, elite donors

**Abbreviations:** NBPGR, National Bureau of Plant Genetic Resources; NARS, National Agricultural Research System; ICARDA, International Centre for Agricultural Research in Dry Areas, PM, Powdery Mildew; FW, Fusarium Wilt; ILWL, International Legume Wild Lentil; PCA, Principle Component Analysis; PC, Principle Component; BIGM, Biodiversity and Integrated Gene Management; CWRs, Crop Wild Relatives; CSKHPAUREC, Chaudhary Sarwan Kumar Hamachal Pradesh Agricultural University Research and Extension Centre; CRBD, Complete Randomized Block Design; UPGMA, Unpaired Group Method Analysis; SMTA, Standard Material Transfer Agreement

**Introduction**

Lentil is a self-pollinating true diploid (2n= 2x=14) grain legume crop having genome size of 4063 Mb

[1]. The cultivated lentil species (*Lens culinaris*) encompasses two groups, established on the basis of distinct morphological characters, the small-seeded (*microsperma*) and large-seeded (*macrosperma*)
[2]. The production and productivity of lentil has increased from an average yield of 565 kg ha^-1^ in 1961-63 to 940 kg ha^-1^ during 2014-2015 [3]. Despite the tremendous improvement, the current lentil yield is much below as compared to other pulses. In India, most of the lentil varieties which were developed through pure line selection showed low yield potential. In cultivated lentil varieties, the yield limiting factors are lack of seedling vigour, very high rate flower drop and low pod setting, lack of lodging resistance and exposure to major biotic and abiotic stresses [4]. The pedigree analysis of some important released varieties exhibited that a few donors have contributed substantially into the background of those varieties [5]. Therefore, to attain further breakthrough for enhancing genetic gains, new target traits are needed to be identified and introgressed into cultivated gene pool for widening the genetic base of cultigens. Crop wild relatives (CWRs) are an invaluable reservoir of productivity enhancement related characters having resilience to climate change and are source of novel traits [6-7]. The global wild lentil germplasm introduced from the International Centre for Agricultural Research in Dry Areas (ICARDA), has been multiplied, characterized and evaluated against target characters [8]. The promising 96 wild lentil accessions selected from 405 global wild collections were validated under multilocational evaluation for identifying stable donors against the target traits. The present study was therefore, undertaken (1) to evaluate promising wild lentil accessions for various target agronomical characters and major biotic stresses and (2) to identify the potential accessions (gene sources) carrying important traits for enhancing genetic gains of cultivated gene pool.

**Materials and methods**

**Plant materials**

The experimental genetic material comprised of 96 wild lentil accessions (Table 1) selected from 405 global wild collections [8] consisting of *L. orientalis* (24), *L. odemensis* (16), *L. tomentosus* (8), *L.* *nigricans* (17), *L. ervoides* (24), *L. lamottei* (5) and *L. culinaris* (2) along with two check varieties (Precoz, and L830). These were evaluated under multi-location and multi-season performance for target characters viz. earliness, pod number, seed yield and resistance against rust, powdery mildew and Fusarium wilt under field and controlled screening conditions. The original identity of these accessions was same as mentioned by the Biodiversity and Integrated Gene Management Unit (BIGM) at ICARDA.

**Agronomic evaluation**

All 96 wild lentil accessions were evaluated under two agro-ecological locations viz. Experimental Farm of NBPGR Regional Station, Shimla (28^0^ 35’ N, 70^0^18’E, altitude 2276 m amsl) during winter season of 2014-2015, 2015-2016 and 2016-2017; and CSKHPKV Mountain Agricultural Research and Extension Centre, Sangla (31^0^ 55’ N, 77^0^ 00 50’ E altitude 3450 m amsl) during summer season of 2015, 2016 and 2017. Each entry was replicated thrice in 3 m long row and 35 cm apart. The observations were recorded against important agro-morphological characters in both locations. Further, numerical data was subjected to statistical analysis using SAS software [9]. The coefficient of variation for various characters was determined using the formulae of Burton [10] and broad sense heritability was also calculated as per the method suggested by Lush [11]. The expected genetic advance was performed as per the procedure of Johnson et al. [12].

**Phenotypic diversity and association study**

The phenotypic diversity analysis was carried out using quantitative data of 96 promising accessions of wild lentil. Euclidean distances were calculated using quantitative data which was used to establish relationship between interspecific accessions. DARwin 5.0 software of Perrier et al. [13] was used for hierarchical clustering of accessions using UPGMA mode based upon Jaccard’s coefficient. Correlation among various parameters was determined using SAS software [9].

**Screening against rust, powdery mildew and Fusarium wilt**

**Rust (*Uromyces*** fabae **(Grev.) Fuckel)**

The experiment was conducted at CSKHPKV Research and Extension Centre, Dhaulakuan (30^0^ 49´ N, 77^0^ 59´E, altitude 468 m amsl) during winter season of 2014-2015, 2015-2016 and 2016-2017. All the test accessions and rust susceptible check ‘var. L830’ were sown in plastic pots (20 cm diameter) filled with a mixture of top field soil and farm yard manure (10:1) in two replications. The pots with plants at pre-flowering stage (~60 days after sowing) of wild accessions were shifted in the field sown with rust susceptible varieties, K 75 and L 830. The test accessions were frequently inoculated by spraying uerdo-acieosporic suspension (1x10^6^ spores/ml) of local isolate of *U. viciae fabae*. The data were recorded on terminal disease reaction using 1–9 rating scales at vegetative and reproductive stages as followed by Mayee and Datar [14].

**Powdery mildew (*Erysiphe trifolii***)

Another experiment was undertaken at CSKHPKV Research and Extension Centre, Dhaulakuan during winter season of 2014-2015, 2015-2016 and 2016-2017. The plants of test accessions were raised in plastic pots in a mixture of soil and farmyard manure mixture (10:1) under green house conditions. The disease symptoms started developing at flower initiation stage and susceptible checks developed disease earlier and faster. The pots with heavily infected susceptible plants were transferred near the test accessions raised in the green house. The heavily infected plants were shaken with the wooden stick to dislodge the conidia which dispersed and infected the healthy plant parts of the test accessions as also suggested by Tiwari et al. [15]. The full infestation of susceptible accessions was observed near green pod stage and observations on terminal disease reaction were recorded at different intervals using 1-9 rating scales [15].

**Fusarium wilt (*Fusarium oxysporum f. sp. lentis* (Vasd. Srin.) Gord)**

All 96 wild lentil accessions were screened against Fusarium wilt resistance at Rafi Ahmad Kidwai Agriculture College, Sehore, Madhya Pradesh (23^0^12’N, 77^0^05’E, altitude 502 m amsl) during winter season of 2014-2015 and 2015-2016. For screening of wild lentil accessions, protocol was standardized as per the method described by Bayaa and Erskine [16-19]. The performance of disease pressure was compared with the resistant (PL639) and susceptible (L-9-12) checks which were

repeated after 15 accessions of each replication. The data were taken using 1-9 rating scales as followed by Bayaa et al. [17, 19].

**Results**

**Frequency distribution of quantitative characters**

The analysis of variance revealed that the wild lentil accessions differed significantly in terms of seed yield plant^-1^ and its important component characters (significant at p=0.05) and also manifested by the range, mean and coefficient of variation for all the metric characters assessed. However, the frequency distribution of important quantitative characters depicted in Fig.1 exhibited that a wide range of variation was observed in plant height, number of branches plant^-1^, number of pods plant^-1^ and seed yield plant^-1^. In plant height, all 96 accessions were grouped into different classes (Fig. 1) and only two accessions ILWL15 (63.50 cm) and ILWL19 (66.50 cm) of *L. nigricans* were reported for maximum height. Likewise, number of branches plant^-1^ was highest in accession ILWL 418 (52 branches) of *L. ervoides* and ILWL 480 (37 branches) of *L. orientalis*. The number of pods plant^-1^ ranged from 3 pods plant^-1^ to 1167 pods plant^-1^ and only two accessions ILWL 418 (1002 pods) of *L.* *ervoides* and ILWL 18 (1167 pods) of *L. nigricans* revealed maximum number. The character seed yield plant^-1^ also showed variation and only two accessions ILWL 18 (13.60g) and ILWL 19 (12.55g) of *L. nigricans* showed maximum seed yield palnt^-1^.

**Diversity analysis**

Wild lentil accessions formed two major clusters I and II in hierarchical clustering as shown in Fig. 2. Major cluster I grouped only three accessions including ILWL418 (*L. ervoides*) from Syria; ILWL18 and ILWL19 (*L. nigricans*) from France and Spain, respectively. Cluster II inhabiting all remaining accessions was further divided into A and B clusters. Bifurcation of Cluster A resulted into two sub clusters AI and AII, , where sub cluster AI was occupied by *L. ervoides* accessions ILWL58 and ILWL292 from Turkey; *L. nigricans* accessions ILWL16 from Alpes Cote d’ Azur and ILWL15 from France; and ILWL14 and ILWL15 of *L. lamottei* from France. Sub cluster AII grouped ILWL7 and ILWL8 of *L. orientalis* from Turkey; ILWL51, ILWL60 and ILWL65 of *L. ervoides* including first from Montenegro and other two from Turkey; and ILWL20 of *L. odemensis* from Palestine. Cluster B was occupied by two sub clusters BI and BII, each of which were further divided into two groups a, b and c, d, respectively. Group d further formed outgroups (d1 and d2) and finally clutches (d2a and

d2b). As Cluster II grouped sub-clusters were occupied by accessions from different wild lentil species belonging to different countries of origin, they showed highest diversity (Supplementary Table 1). Euclidean dissimilarity matrices for core set accessions varied from 6.01 between ILWL429 (*L.* *lamottei*) from Spain and ILWL90 (*L. tomentosus*) from Turkey to 2156.52 between ILWL18 (*L. nigricans*) from France and ILWL476 (*L. orientalis*) from Turkey (Supplementary Table 1). First two principal coordinates in factorial analysis explained 99.07 % and 0.52 % variability, respectively. In both type of groupings, the role of geographical or country of origin of accessions was not observed.

**Correlations among different parameters**

The correlation coefficient among different agro-morphological traits of wild lentil accessions is shown in Table 2. Correlation indices highlighted a number of significant inter-relationships (both +ve and -ve) among studied traits. In correlation studies, when observations were large in number, many correlations become significant due to increase in test power. So in the present study, correlations which had >0.4 r-values were considered. Significant positive correlation was observed between days to flowering and maturity. Similarly, significant positive correlations were obtained between seed yield plant^-1^, biological yield plant^-1^ and harvest index. These traits also showed positive correlations with plant height (r^2^ <4.0 in case of biological yield plant^-1^), number of branches plant^-1^ (r^2^ <4.0 in case of harvest index), number of pods plant^-1^ and seeds plant^-1^. Seeds plant^-1^ showed significant correlation with plant height, branches plant^-1^ and pods plant^-1^. The number of pods plant^-1^ exhibited significant positive correlation with branches plant^-1^. Significant negative correlation was observed between days to flowering and seed yield plant^-1^, seeds plant^-1^, pods plant^-1^, and harvest index.

**Principle component analysis**

The variability pattern among wild lentil accessions and the contribution of different traits towards these patterns were determined using Principal Component Analysis (PCA). Maximum variability (66.31%) was explained by only three components with Eigen value >1.0 (Table 3). Of the total variation, 42.37% was exhibited by PC1, and the contributing traits with high coefficients included seed yield plant^-1^, seeds plant^-1^, pods plant^-1^, harvest index and biological yield plant^-1^. Similarly, 15.04% variability was explained by PC2, and the contributing traits with high coefficients included days to maturity, days to flowering, rust incidence and plant height. Similarly, PC3 contributed 8.89% of overall variability, and the traits contributing to diversity included 100- seed weight, Fusarium wilt

and Powdery mildew incidence. Fig. 3a and 3b shows the two dimensional variability pattern and contributing traits towards variability, respectively.

**Screening against major biotic stresses**

**Rust resistance (*Uromyces fabae* (Grev.) Fuckel)**

Majority of wild lentil accessions were resistant and moderately resistant against rust under controlled artificial inoculation conditions. Accessions ILWL90, ILWL195, ILWL198 and ILWL480 (*L.* *tomentosus*); ILWL230, 349, 476 (*L. orientalis*); ILWL203, 235 and 357 (*L. odemensis*); ILWL60, 204, 292, 414 and 442 (*L. ervoides*); and ILWL16, 18, 37, 460 and EC718266 (*L. nigricans*) were identified as resistant (Table 4 and 5). The remaining accessions were scored as moderately resistant, susceptible and some of them were highly susceptible against the pathogen. The distribution of accessions into various reaction categories based on disease score is presented in Fig. 4.

**Powdery mildew resistance (*Erysiphe trifolii*)**

Out of 24 accessions of *L. orientalis*, ILWL 230 and ILWL 476 were found highly resistant. Likewise, in *L. odemensis*, accessions ILWL 39, ILWL 203, and IG 136788; ILWL 198 and ILWL 480 of *L.* *tomentosus* were also found to be highly resistant (Table 4 and 5). However, accessions ILWL 51, ILWL 401, ILWL 418 and ILWL 441 of *L. ervoides*; ILWL9, ILWL22, ILWL34, ILWL37 and ILWL191 of *L. nigricans*; and ILWL 29 of *L. lamottei* with disease reaction 2 were found to be resistant. The remaining accessions were either moderately resistant or susceptible against the pathogen (Fig. 5).

**Fusarium wilt resistance (*Fusarium oxysporum f. sp. lentis* (Vasd. Srin.) Gord)**

The wild lentil accessions, which showed resistant disease reaction to Fusarium wilt included ILWL7, ILWL96, ILWL117, ILWL227, ILWL246, ILWL344 and ILWL359 (*L. orientalis*); ILWL15 and ILWL39 (*L. odemensis*); ILWL199 and ILWL 308 (*L. tomentosus*)*.* Accessions ILWL58, ILWL398, ILWL414, IG136626 (*L. ervoides*); ILWL17, ILWL19, ILWL23, ILWL34, ILWL38 and ILWL474 (*L. nigricans*) and ILWL20 and ILWL430 (*L. lamottei*) were found resistant against the wilt (Table 4 and 5). Some accessions were moderately resistant and the remaining was susceptible against the pathogen as depicted in Fig. 6.

**Discussion**

The precise evaluation of crop wild relatives is pre-requisite to identify target traits of interest followed by their introgression into the background of cultivated varieties for enhancing genetic gains [20-22]. A wide range of variation in wild lentil accessions was observed against the target traits viz. earliness, high pod number and seed yield as also shown by the coefficient of variation, thereby suggesting diverse genetic makeup and geographical origins of wild lentil collections included in the present study. These promising accessions belonging to different taxa and ecological niches could be useful resource for enhancing genetic gains of cultivated varieties. Accessions ILWL 18 and ILWL 19 of *L. nigricans* were found promising for high seed yield plant^-1^, suggesting their broader genetic base as also reflected in clustering pattern of wild accessions based on quantitative data analysis. Euclidean dissimilarity matrices of wild lentil accessions ranged from 6.01 to 2156.52, which revealed very high variability among interspecific lentil taxa. The observed variability could be due to the presence of diverse origins of these wild accessions. Among the different countries of origin, accessions from Turkey showed maximum variability for the studied traits suggesting more exploration and collection of lentil germplasm. Viera et al. [23] characterized wheat germplasm based on phenotypic traits and obtained distances up to 196.61. The report of existing variability among germplasm accessions provides an idea about the expected heterosis for starting breeding programme. A broad range of Euclidean distances obtained in the present study shows the importance of wild lentil accessions as a source of diverse heterotic material [24]. However, the diversity pattern further suggested that no role was played by the geographical distributions of global accessions in their grouping. This grouping pattern can be described on the basis of different genetic constitution of accessions. Further, it might be due to continuous gene flow occurrence among wild lentil taxa. No role of geographical distributions was observed in grouping pattern of accessions clustered on the basis of morphological, biochemical and molecular markers in guar [25]. Similar observation in wild lentil accessions was also reported by Singh et al. [8] and Kumar et al. [26]. Further, significant positive correlation of seed yield plant^-1^ with seeds plant^-1^, pods plant^-1^, branches plant^-1^ was observed in lentil accessions and similar correlations have also been reported by other workers [27-29]. They reported that seeds plant^-1^, biological yield plant^-1^, pods plant^-1^, and harvest index are the important yield component traits in lentil contributing towards yield enhancement. Such correlations are useful for lentil genetic improvement and breeding programmes. These observations were found to be in accordance with the results of Toklu et al. [29] and Abo-Hegazy et al. [30]. The traits which contributed heavily towards

variability included seed yield plant^-1^, seeds pod^-1^, pods plant^-1^, biological yield plant^-1^ and harvest index. These findings highlighted the significance of yield related component characters generating substantial variations in wild lentil accessions.

Furthermore, wild taxa of lentil are known to be resistant against major biotic and abiotic stresses [8]. They are the carrier of important characters and have potential value in diversification of cultivated gene pool for enhancing genetic gains. In the promising experiments on screening of wild lentil accessions against major biotic stresses, development of severe disease symptoms on the susceptible cultivars were clearly evident in the real field conditions, indicating the effectiveness of inoculation procedures. Some wild lentil accessions revealed resistant disease reaction against more than one disease (Table 5) viz. accessions ILWL230 and ILWL476 of *L. orientalis* (rust and powdery mildew); accessions ILWL9 and ILWL37 of *L. nigricans* (rust and powdery mildew); accession IG136639 of *L. ervoides* (powdery mildew and Fusarium wilt) and accession ILWL308 of *L.* *tomentosus* (rust and Fusarium wilt). It is important to mention that the northwestern part has been the traditional lentil cultivation area in India. But in late 1980s, among various factors including the epidemics of rust, powdery mildew and Fusarium wilt resulted in drastic reduction of lentil area and production as well. In this context, gene sources identified against rust, powdery mildew and *Fusarium* wilt in the present study can prove to be useful donors which could be exploited through pre-breeding with the aim to develop usable germplasm for developing resistant lentil varieties, especially for the epidemiologically important Indian conditions. Furthermore, the development of lentil cultivars with combined resistance against major biotic stresses may help in the development of appropriate management strategies in future in conjunction with allelism studies to identify and pyramid novel genes for resistance breeding of lentil.

**Conclusions**

During the past few years, little efforts were made on evaluation of wild lentil genetic resources in India. However, this is the first attempt on characterization and evaluation of all available global wild lentil germplasm against the target traits of interest under diverse agro-ecological conditions. Further, the study has helped in identifying the confirmed and stable gene sources (donors) across intra and interspecific accessions viz. ILWL203 (EC789113) of *L. odemensis* for rust and high pod number; ILWL230 (EC718515), ILWL476 (EC718605) of *L. orientalis* for rust and powdery mildew; ILWL191 (EC718682), ILWL9, and ILWL37 of *L. nigricans* for rust and powdery mildew, IG136639 of *L. ervoides* for powdery mildew and Fusarium wilt and ILWL308 (EC728782) of *L. tomentosus* for

rust and Fusarium wilt. These gene sources could be taken in lentil wide hybridization programme for

enhancing	genetic	gains	of	cultivated	varieties	and	could	also	be	shared	among	lentil

breeders/researchers	in	the	country or	elsewhere	under	Standard	Material	Transfer	Agreement

(SMTA) for strengthening the on-going lentil genetic improvement programmes.

**Acknowledgements**

We thank the Head, Gene Bank at ICARDA, Aleppo Syria for providing all available wild lentil

accessions to Indian National Gene Bank. The authors also thank the Department of Agriculture

Cooperation and Farmer’s Welfare, Govt. of India for grant in aid to accomplish the proposed

objectives.

**References**

1. Arumuganathan K., Earle ED. Nuclear DNA content of some important plant species. Plant Mol. Biol Rep*.*1991; 9: 208–218.
2. Ferguson ME, Maxted N, Slageren MV, Robertson LD. A re-assessment of the taxonomy of Lens. Bot. J. Linn. Soc. 2000; 133: 41–59.
3. Food and Agriculture Organization (FAO) FAO Statistical database of the United Nations, Food and Agriculture Organization Rome; 2015.
4. Erskine W, Muehlbauer FJ, Sarker A, Sharma B. An Introduction, In: W. Erskine, F.J.

Muehlbauer, A. Sarker and B. Sharma: The lentil, botany, production and uses. CAB International; 2009 p.1-3.

5. Kumar S, Gupta S, Chandra S, Singh BB. How wide is the genetic base of pulse crops, Pulses in New Perspective In: Ali M, Singh BB, Kumar S, Dhar V eds. Indian Society of Pulses Research and Development, Indian Institute of Pulses Research, Kanpur India; 2003.
6. Maxted N, Ford BV, Kell SP, Iriondo JM, Dulloo ME. Crop wild relative conservation and use, Wallingford, UK, CABI publishing; 2008.
7. Lane A, Jarvis A. Changes in climate will modify the geography of crop suitability: Agricultural biodiversity can help with adaptation. Climate Proofing Innovation for Poverty Reduction and Food Security, ICRISAT, Patancheru; 2007.
8. Singh M, Bisht IS, Kumar S, Dutta M, Bansal KC, Sarker A, Amri A, Kumar S, Datta SK. Global wild annual Lens collection: a potential resource for lentil genetic base broadening and yield enhancement. PLoS ONE, (2014) 10.1371/journal.pone.0107781
9. SAS. Sas Institute Inc. Cary, NC, SAS Institute; 2011.
10. Burton GW. Quantitative inheritance in grasses, in Proceedings of the 454 International Grassland Congress, State College, PA: Pennsylvania State College; 1952.
11. Lush JL. Intrusive collection of regression of offspring on dams as a method 501 of estimating heritability of characters. Proc. Am. Soc. Anim. Prod. 1940; 33: 293–301.
12. Johnson HW, Robinson HF, Comstock RE. Estimates of genetic and environmental variability in Soybean. Agron. 1955; J. 47: 314–318.

13. Perrier X, Flori A, Bonnot F. Data analysis methods, In: Hamon, P., Seguin, M., Perrier, X., Glaszmann, J.C. Ed., Genetic diversity of cultivated tropical plants. Enfield, Science Publishers Montpellier; 2003.
14. Mayee CD, Datar VV. Phytopathometry. Technical Bulletin-1, Marathwada Agricultural University, Parbhani; 1986.
15. Tiwari KR, Penner GA, Warkentin TD, Rashid KY. Pathogenic variation in Erisiphe polygoni, the casual organism of powdery mildew of lentil. Can. J. Plant Pathol. 1997; 19: 267–271.
16. Bayaa B, Erskine W. A screening technique for resistance to vascular wilt in lentil. Arab J. Plant Prot. 1990; 8: 30–33.
17. Bayaa B, Erskine W, Hamdi A. Evaluation of wilt lentil collection for resistance to vascular wilt. Genet. Resour. Crop Evol. 1995; 42: 231–235.
18. Bayaa B, Erskine W, Singh M. Screening lentil for resistance to Fusarium wilt: methodology

and sources of resistance. Euphytica. 1997; 98: 69–74.

19. Eujayl I, Erskine W, Bayaa B, Baum M, Pehu E. Fusarium vascular wilt in lentil: inheritance

and identification of DNA markers for resistance. Plant Breed. 1998; 117: 497–499.

20. Duvick DN. Genetic diversity in major farm crops on the farm and in reverse. Eco. Bot. 1984;
    38: 151–178.
21. Lazaro A, Ruiz M, Rosa L, Martin I. Relationship between agro-morphological characters and climate parameters in Spanish landraces of lentil. Genet Resour Crop Evol. 2001; 48: 239-249.
22. Naghavi MR, Jahansouz MR. Variation in the agronomic and morphological traits of lentil accessions. J. Integ. Plant Biol. 2005; 47: 375–379.
23. Vieira EA, Carvalho FIF, Bertan de I, Kopp M, Zimmer PD. Association between genetic distances in wheat as estimated by AFLP and morphological markers. Genet. Mol. Biol. 2007;
    30: 392–399.
24. Gupta SK, Nepolean T, Shaikh CG, Rai K, Hash CT, Das RR, Rathore A. Phenotypic and molecular diversity-based prediction of heterosis in pearl millet (Pennisetum glaucum L. (R.) Br.).The Crop Journal. 2018; 6: 271-281.
25. Kumar S, Joshi UN, Singh V, Singh JV, Saini ML. Characterization of released and elite genotypes of guar from Indian proves unrelated to geographical origin. Genet Resour Crop Evol. 2013; 60: 2017-2032.
26. Kumar S, Choudhary AK, Rana KS Sarker A, Singh M. Biofortification potential of global

wild annual lentil core collection. PLOS ONE. 2018; [https://doi.org/10.1371/journal.pone.0191122.](https://doi.org/10.1371/journal.pone.0191122)

27. Luthra SK, Sharma PC. Correlation and path analysis in lentil. Lens New Letter. 1990; 7: 5-8.
28. Sarwar G, Abbas G, Asghar MJ. Quantitative analysis of yield related traits in lentil. J. Agric. Res. 2013; 51: 239-246.
29. Toklu F, Ozkan H, Karakoy T, Coyne C. Evaluation of advanced lentil lines for diversity in seed mineral concentration, grain yield and yield components. J Agric. Sci. 2015; 23: 213-222.
30. Abo-Hegazy SRE, Selim T, El-Emam EAA. Correlation and path co-efficient analysis of yield and some yield components in lentil. Egyptian J. Pl. Breed. 2012; 16: 147-159.

31. Wong MM, Gujaria N, Ramsay L, Yuan HY, Caron C, Diapari M, Vanderberg A, Bett KE. Classification and characterization of species within the genus *Lens* using genotyping-by-sequencing. PLoS ONE. 2015; 10(3) e0122025.

**Table 1: List of taxa wise wild lentil accessions along with their country origin**

| **S.** | **Taxa/accession** | | **Origin** | **S. No.** | **Taxa/accession** | | **Country origin** |
| --- | --- | --- | --- | --- | --- | --- | --- |
| **No** |  |  |  |  |  |  |  |
|  | ***L. orientalis*** | |  |  | ***L. lamottei*** |  |  |
| 1 | ILWL 7 | (EC718234) | Turkey | 50 | ILWL 14 | (EC718236) | France |
| 2 | ILWL 8 | (EC718235) | Turkey | 51 | ILWL 15 | (EC718238) | France |
| 3 | ILWL 75 (EC718456) | | Israel | 52 | ILWL 29 | (EC718251) | Spain |
| 4 | ILWL 89 | (EC718470) | Turkey | 53 | ILWL 429 (EC718686) | | Spain |
| 5 | ILWL 95 | (EC718475) | Turkey | 54 | EC718692 |  | France |
| 6 | ILWL 96 | (EC718476) | Turkey |  | ***L.* o*demensis*** | |  |
| 7 | ILWL 101 | (EC718479) | Turkey | 55 | ILWL 20 | (EC718243) | Palestine |
| 8 | ILWL 117 (EC718488) | | Syria | 56 | ILWL 35 | (EC718276) | Turkey |
| 9 | ILWL 124 (EC718490) | | Syria | 57 | ILWL 39 | (EC718277) | Palestine |
| 10 | ILWL 181 (EC718491) | | Syria | 58 | ILWL 165 (EC718281) | | Syria |
| 11 | ILWL 227 (EC718513) | | Syria | 59 | ILWL 166 (EC718282) | | Syria |
| 12 | ILWL 230 (EC718515) | | Syria | 60 | ILWL 167 (EC718283) | | Syria |
| 13 | ILWL 243 (EC718519) | | Syria | 61 | ILWL 203 (EC789113) | | Syria |
| 14 | ILWL 246 (EC718521) | | Syria | 62 | ILWL 235 (EC718291) | | Syria |
| 15 | ILWL 278 (EC718529) | | Turkey | 63 | ILWL 320 (EC718295) | | Turkey |
| 16 | ILWL 330 (EC718548) | | Syria | 64 | ILWL 357 (EC718297) | | Syria |
| 17 | ILWL 343 (EC718554) | | Syria | 65 | ILWL 361 (EC718298) | | Syria |
| 18 | ILWL 344 (EC718555) | | Syria | 66 | ILWL 409 (EC718301) | | Syria |
| 19 | ILWL 349 ( EC718560) | | Syria | 67 | ILWL 436 (EC718302) | | Turkey |
| 20 | ILWL 359 (EC718566) | | Syria | 68 | ILWL 438 (EC718303) | | Turkey |
| 21 | ILWL 384 (EC718585) | | Tajikistan | 69 | EC718311 |  | Israel |
| 22 | ILWL 443 (EC718596) | | Turkey | 70 | EC718694 |  | Syria |
| 23 | ILWL 476 (EC718605) | | Turkey |  | ***L. ervoides*** |  |  |
| 24 | ILWL 480 (EC718609) | | Syria | 71 | EC718692 |  | Spain |
|  | ***L. nigricans*** | |  | 72 | ILWL 43 | (EC718316) | Croatia |
| 25 | ILWL 9 | (EC718236) | Syria | 73 | ILWL 50 | (EC718321) | Croatia |
| 26 | ILWL 14 | (EC718267) | Syria | 74 | ILWL 51 | (EC718322) | Montenegro |
| 27 | ILWL 15 | (EC718238) | France | 75 | ILWL 58 | (EC718329) | Turkey |
| 28 | ILWL16 | (EC718239) | Alpes-Cote |  |  |  |  |
|  |  |  | d'Azur | 76 | ILWL 60 | (EC718331) | Turkey |
| 29 | ILWL 18 | (EC718241) | France | 77 | ILWL 61 | (EC718332) | Turkey |
| 30 | ILWL 19 | (EC718242) | Spain | 78 | ILWL 63 | (EC718333) | Turkey |
| 31 | ILWL 22 | (EC718245) | Italy | 79 | ILWL 65 | (EC718335) | Turkey |
| 32 | ILWL 31 | (EC718254) | Spain | 80 | ILWL 66 | (EC718336) | Turkey |
| 33 | ILWL 34 | (EC718256) | Ukraine | 81 | ILWL 204 (EC718360) | | Turkey |
| 34 | ILWL 37 | (EC718257) | Turkey | 82 | ILWL 234 (EC718362) | | Syria |
| 35 | ILWL 38 | (EC718258) | Turkey | 83 | ILWL 269 (EC718370) | | Turkey |
| 36 | ILWL 191 (EC718682) | | Croatia | 84 | ILWL 276 (EC718374) | | Turkey |
| 37 | ILWL 460 (EC718262) | | Turkey | 85 | ILWL 292 (EC718378) | | Turkey |
| 38 | EC718266 | | Italy | 86 | ILWL 321 (EC718383) | | Turkey |
| 39 | EC718270 | | Croatia | 87 | ILWL 398 (EC718401) | | Lebanon |
| 40 | EC718273 | | Spain | 88 | ILWL 401 (EC718404) | | Lebanon |
| 41 | EC718275 | | Turkey | 89 | ILWL 408 (EC718407) | | Syria |
|  | ***L. tomentosus*** | |  | 90 | ILWL 414 (EC718411) | | Syria |
| 42 | ILWL 90 | (EC718471) | Turkey | 91 | ILWL 418 (EC718413) | | Syria |
| 43 | ILWL 195 (EC718682) | | Syria | 92 | ILWL 441 (EC718417) | | Turkey |
| 44 | ILWL 198 (EC718685) | | Syria | 93 | ILWL 442 (EC718418) | | Turkey |
| 45 | ILWL 199 (EC718686) | | Syria | 94 | EC718439 |  | Israel |
| 46 | ILWL 305 | (EC728782) | Turkey |  | ***L. culinaris*** | |  |
| 47 | ILWL 308 | (EC728782) | Turkey | 95 | ILL 8006 |  | Syria |
| 48 | ILWL 480 | (EC741251) | Syria | 96 | ILL 10829 |  | Syria |
| 49 | EC718673 | | Syria |  |  |  |  |

**Table2: Correlations among various agro-morphological traits of wild lentil accessions**

| **Traits** | **DF** | **DM** | **PH** | **NBPP** | **NPPP** | **NSPPL** | **NSPPD** | **SDWT** | **SYPP** | **BYPP** | **HI** | **RT** | **PM** | **FW** |
| --- | --- | --- | --- | --- | --- | --- | --- | --- | --- | --- | --- | --- | --- | --- |
| **DF** | 1.00 | .750(**) | -0.13 | -.219(*) | -.542(**) | -.534(**) | -0.16 | -0.06 | -.495(**) | - | -.543(**) | -0.15 | -0.06 | -0.15 |
|  |  |  |  |  |  |  |  |  |  | .311(**) |  |  |  |  |
|  |  |  |  |  |  |  |  |  |  |  |  |  |  |  |
| **DM** | .750(**) | 1.00 | 0.04 | -0.15 | -.346(**) | -.319(**) | -.256(*) | -0.09 | -.350(**) | -.235(*) | -.319(**) | -0.02 | 0.03 | -0.17 |
|  |  |  |  |  |  |  |  |  |  |  |  |  |  |  |
| **PH** | -0.13 | 0.04 | 1.00 | .242(*) | .360(**) | .411(**) | -0.11 | -0.15 | .468(**) | .344(**) | .438(**) | .342(**) | 0.15 | -0.14 |
|  |  |  |  |  |  |  |  |  |  |  |  |  |  |  |
| **NBPP** | -.219(*) | -0.15 | .242(*) | 1.00 | .544(**) | .536(**) | -.285(**) | -0.09 | .504(**) | .539(**) | .388(**) | .328(**) | .272(**) | 0.08 |
|  |  |  |  |  |  |  |  |  |  |  |  |  |  |  |
| **NPPP** | -.542(**) | -.346(**) | .360(**) | .544(**) | 1.00 | .987(**) | -0.15 | -0.09 | .928(**) | .833(**) | .738(**) | .367(**) | .290(**) | 0.03 |
|  |  |  |  |  |  |  |  |  |  |  |  |  |  |  |
| **NSPPL** | -.534(**) | -.319(**) | .411(**) | .536(**) | .987(**) | 1.00 | -0.18 | -0.08 | .956(**) | .820(**) | .788(**) | .308(**) | .322(**) | -0.02 |
|  |  |  |  |  |  |  |  |  |  |  |  |  |  |  |
| **NSPPD** | -0.16 | -.256(*) | -0.11 | -.285(**) | -0.15 | -0.18 | 1.00 | -0.12 | -.244(*) | -.247(*) | -0.16 | -.291(**) | -.224(*) | 0.13 |
|  |  |  |  |  |  |  |  |  |  |  |  |  |  |  |
| **SDWT** | -0.06 | -0.09 | -0.15 | -0.09 | -0.09 | -0.08 | -0.12 | 1.00 | 0.00 | -0.12 | 0.02 | -0.02 | 0.07 | 0.14 |
|  |  |  |  |  |  |  |  |  |  |  |  |  |  |  |
| **SYPP** | -.495(**) | -.350(**) | .468(**) | .504(**) | .928(**) | .956(**) | -.244(*) | 0.00 | 1.00 | .814(**) | .841(**) | .373(**) | .391(**) | 0.00 |
|  |  |  |  |  |  |  |  |  |  |  |  |  |  |  |
| **BYPP** | -.311(**) | -.235(*) | .344(**) | .539(**) | .833(**) | .820(**) | -.247(*) | -0.12 | .814(**) | 1.00 | .508(**) | .377(**) | .283(**) | -0.02 |
|  |  |  |  |  |  |  |  |  |  |  |  |  |  |  |
| **HI** | -.543(**) | -.319(**) | .438(**) | .388(**) | .738(**) | .788(**) | -0.16 | 0.02 | .841(**) | .508(**) | 1.00 | .312(**) | .347(**) | 0.00 |
|  |  |  |  |  |  |  |  |  |  |  |  |  |  |  |
| **Rust** | -0.15 | -0.02 | .342(**) | .328(**) | .367(**) | .308(**) | -.291(**) | -0.02 | .373(**) | .377(**) | .312(**) | 1.00 | .314(**) | -0.08 |
|  |  |  |  |  |  |  |  |  |  |  |  |  |  |  |
| **PM** | -0.06 | 0.03 | 0.15 | .272(**) | .290(**) | .322(**) | -.224(*) | 0.07 | .391(**) | .283(**) | .347(**) | .314(**) | 1.00 | -0.02 |
|  |  |  |  |  |  |  |  |  |  |  |  |  |  |  |
| **FW** | -0.15 | -0.17 | -0.14 | 0.08 | 0.03 | -0.02 | 0.13 | 0.14 | 0.00 | -0.02 | 0.00 | -0.08 | -0.02 | 1.00 |
|  |  |  |  |  |  |  |  |  |  |  |  |  |  |  |

*Correlation is significant at the 0.05 level, **Correlation is significant at the 0.01 level

DF, Days to flowering; DM, Days to maturity; PH, Plant height; NBPP, Number of branches plant^-1^; NPPP, Number of pods plant^-1^; NSPPL, Number of seeds plant^-1^; NSPPD, Number of seeds pod^-1^; SDWT, Seed weight; SYPP, Seed yield plant; BYPP, Biological yield plant; HI, Harvest index; RT, Rust; PM, Powdery mildew; FW, Fusarium wilt

**Table 3: Eigen vectors, eigen values, individual and cumulative percentages of variation explained by the first three principal components (PC) of wild lentil accessions**

| **Traits** | **Prin1** | **Prin2** | **Prin3** |
| --- | --- | --- | --- |
| Days to flowering | -0.25 | 0.49 | 0.00 |
| Days to maturity | -0.19 | 0.56 | -0.01 |
| Plant height (cm) | 0.20 | 0.22 | -0.30 |
| Number of branches plant^-1^ | 0.25 | 0.12 | 0.09 |
| Number of pods plant^-1^ | 0.39 | -0.05 | -0.06 |
| Number of seeds plant^-1^ | 0.39 | -0.02 | -0.06 |
| Number of seeds pod^-1^ | -0.10 | -0.41 | -0.37 |
| 100 -seed weight (g) | -0.02 | -0.09 | 0.71 |
| Seed yield plant^-1^ (g) | 0.40 | 0.04 | 0.02 |
| Biological yield plant^-1^ (g) | 0.34 | 0.10 | -0.06 |
| Harvest index (%) | 0.35 | -0.04 | 0.02 |
| Rust | 0.26 | 0.26 | 0.04 |
| Powdery mildew | 0.17 | 0.22 | 0.33 |
| Fusarium wilt | 0.00 | -0.26 | 0.37 |
| Eigen value | 5.93 | 2.11 | 1.24 |
| Percent | 42.37 | 15.04 | 8.89 |
| Cum. Percentage | 42.37 | 57.42 | 66.31 |

**Table: 4. Range of variation of wild lentil accessions against the pathogens of rust, powdery mildew and *Fusarium* wilt**

| **Species** |  | **Rust** |  |  | **Powdery mildew** |  |  | **Fusarium wilt** | |  |
| --- | --- | --- | --- | --- | --- | --- | --- | --- | --- | --- |
|  | **Range** | **Mean ±SE** | **CV (%)** | **Range** | **Mean ±SE** | **CV (%)** | **Range** | **Mean ±SE** |  | **CV (%)** |
| ***L. culinaris*** | 1-1 | 1.00±0.00 | 0.00 | 0-5 | 5.00±0.00 | 0.00 | 1-7 | 4.00±3.00 |  | 106.07 |
| ***L. orientalis*** | 1-9 | 3.41±0.70 | 84.23 | 0-5 | 3.56±0.35 | 42.29 | 1-9 | 4.44±0.74 |  | 70.40 |
| ***L. tomentosus*** | 1-5 | 1.86±0.59 | 84.73 | 0-5 | 2.29±0.75 | 86.45 | 1-9 | 5.00±1.15 |  | 61.10 |
| ***L. odemensis*** | 1-6 | 2.33±0.40 | 66.13 | 0-4 | 1.60±0.39 | 93.90 | 1-9 | 5.67±0.64 |  | 43.57 |
| ***L. ervoides*** | 1-8 | 1.95±0.42 | 92.93 | 0-5 | 1.90±0.45 | 105.12 | 1-9 | 5.18±0.72 |  | 65.14 |
| ***L. nigricans*** | 1-7 | 2.10±0.46 | 98.81 | 0-5 | 2.52±0.52 | 95.11 | 1-9 | 4.43±0.69 |  | 71.61 |
| ***L. lamottei*** | 1-7 | 3.43±0.97 | 75.04 | 0-5 | 3.29±0.75 | 60.14 | 1-9 | 4.43±1.04 |  | 62.33 |

**Table 5: Identification of donor accessions for their introgression to lentil genetic improvement against important agronomic and major biotic stress related traits**

| **Taxa/ trait** | **Accession** |  | **Gene pool** | **Country origin** |
| --- | --- | --- | --- | --- |
| **Agronomic** |  |  |  |  |
| *L. orientalis* | ILWL255, 476 ((High pods plant^-1^, shorter | internode) | Primary | Turkey, Syria |
| *L. odemensis* | ILWL203, 237 (High pods plant^-1^, shorter | internode) | Primary | Syria |
| *L. ervoides* | ILWL294, 321 ((High pods plant^-1^, shorter | internode) | Tertiary | Turkey, Syria |
| **Rust** |  |  |  |  |
| *L. orientalis* | ILWL230, 349, 466 |  | Primary | Turkey |
|  |  |  |  |  |
| *L. odemensis* | ILWL235, 357, ILWL203 |  | Primary | Syria |
| *L. tomentosus* | ILWL 90, 95,198, 308, 480 |  | Primary | Syria |
| *L. ervoides* | ILWL60, 92, 269, 292, 414, 419, IG136226 | | Tertiary | Turkey, Syria |
| *L. nigricans* | ILWL9, 17, 37, 111, 460, IG136639, IG136648 | | Quaternary |  |
| **Powdery mildew** |  |  |  |  |
| *L. orientalis* | ILWL 230, 466 |  | Primary | Syria |
| *L. odemensis* | ILWL 39, 196, IG136788 |  | Primary | Turkey, Syria |
| *L. tomentosus* | ILWL 198, 480 |  | Primary | Turkey, Syria |
| *L. ervoides* | ILWL51, 401, 418, 441 |  | Tertiary | Montenegro, Lebanon, |
|  |  |  |  | Syria, Turkey |
| *L. nigricans* | ILWL6, 9, 22, 34, 37, 111 |  | Quaternary | Turkey, Syria Italy, |
|  |  |  |  | Ukraine, Turkey, Turkey |
| *L. lamottei* | ILWL 29 |  | Secondary | Spain |
| **Fusarium wilt** |  |  |  |  |
| *L. orientalis* | ILWL7, 96, 117, 227, 246, 344, 359 |  | Primary | Turkey, Turkey, Syria, |
|  |  |  |  | Syria, Syria, Syria, Syria, |
| *L. odemensis* | ILWL15, 39 |  | Primary | France, Turkey |
| *L. tomentosus* | ILWL 199, 308 |  | Primary |  |
| *L. ervoides* | ILWL 58, 398, 414, IG136626 |  | Tertiary | Turkey , Lebanon, Syria |
|  |  |  |  | Israeli |
| *L. nigricans* | ILWL17, 19, 23, 34, 38, 474 |  | Quaternary | Spain, Italy, Ukraine, |
|  |  |  |  | Turkey, Turkey |
| *L. lamottei* | ILWL 20, 430 |  | Secondary | PAL, Spain, |

Gene pool classification of Wong et al. [31]

Manuscript

[Click here to access/download;Manuscript;PLOS ONE-Edited](https://www.editorialmanager.com/pone/download.aspx?id=25794312&guid=ae9040b0-f307-40e9-825a-7b0713be15fa&scheme=1) [manuscript-using track chnage.doc](https://www.editorialmanager.com/pone/download.aspx?id=25794312&guid=ae9040b0-f307-40e9-825a-7b0713be15fa&scheme=1)

**Evaluation and identification of wild lentil accessions for enhancing genetic gains of cultivated varieties**

Mohar Singh^1^*, Sandeep Kumar^2^, Ashwani Kumar Basandrai^3^, Daisy Basandrai^3^, Nikhil Malhotra^1^, **Formatted:** Superscript Deep Rattan Saxena^4^, Dorin Gupta^5^, Ashutosh Sarker^6^ and Kuldeep Singh^2^

1. National Bureau of Plant Genetic Resources Regional Station, Shimla-171 004, India
2. National Bureau of Plant Genetic Resources, Pusa New Delhi, 110 012, India
3. CSK Himachal Pradesh Agricultural University Palampur-176 062, India
4. Rafi Ahmad Kidwai, College of Agriculture Sehore, India
5. Faculty of Veterinary and Agricultural Sciences, The University of Melbourne, Melbourne, Australia
6. South Asia and China Regional Programme of International Centre for Agricultural Research in Dry Area, DPS Marg, Pusa New Delhi-110 012, India

*Corresponding author

Dr Mohar Singh

ICAR-NBPGR Regional Station Shimla- 171 004, India

**Abstract**

~~The d~~Domesticated lentil ~~species~~ has a relatively narrow genetic base ~~worldwide~~ globally and ~~majority~~ ~~of~~most released varieties are susceptible to ~~important~~ severe biotic and abiotic stresses. The crop wild realtives could provide new traits of interest for tailoring novel germplasm and cultivated lentil improvement~~in cultivated varieties~~. The ~~prime aim of this research~~primary objective of this study was to evaluate ~~accessions of~~ wild lentil accessions for ~~identifying~~ identification of ~~target~~ economically viable agro-morphological traits and resistance against major biotic stresses. The study has revealed substantial variations in seed yield and its important component characters. Further, the diversity analysis of wild accessions ~~exhibited~~ showed two major clusters ~~and further~~which were bifurcated into sub-clusters, thereby suggesting their wider genetic distance divergence. of from wider ~~large~~ ~~accessions of~~ *~~L. nigricans~~* ~~and~~ *~~L. ervoides~~* ~~taxa.~~ However, principal component analysis ~~also~~ revealed that seed yield plant^-1^, number of seeds plant^-1^, number of pods plant^-1^, harvest index and biological yield plant^-1^ contributed significantly to the total genetic variation assessed in wild lentil ~~species~~taxa. Moreover, ~~S~~some of the wild accessions collected from Syria and Turkey regions showed resistance against more than one disesase indicatinged rich diversity of lentil genetic resources. The identification of most promising genotypes carrying resistance against major biotic stresses could be ~~promoting their utilization~~utilized in~~to~~ the cultivated or susceptible varieties of lentil for enhancing genetic gains. ~~Some of wild accessions showed resistance against more than one disease, were~~ ~~collected from Syria and Turkey regions, indicating rich diversity of lentil genetic resources.~~ The study has also identified some trait specific accessions, which could be taken into the consideration~~,~~ while planning distant hybridization in lentil ~~for tailoring new germplasmbreeding programmes.~~

**Key words**: wild lentil, germplasm enhancement, agronomic evaluation, biotic stresses, elite donors

**Abbreviations:** NBPGR, National Bureau of Plant Genetic Resources; NARS, National Agricultural Research System; ICARDA, International Centre for Agricultural Research in Dry Areas, PM, Powdery Mildew; FW, Fusarium Wilt; ILWL, International Legume Wild Lentil; PCA, Principle Component Analysis; PC, Principle Component; BIGM, Biodiversity and Integrated Gene Management; CWRs, Crop Wild Relatives; CSKHPAUREC, Chaudhary Sarwan Kumar Hamachal Pradesh Agricultural University Research and Extension Centre; CRBD, Complete Randomized Block Design; UPGMA, Unpaired Group Method Analysis; SMTA, Standard Material Transfer Agreement

**Introduction**

Lentil is a~~an autogamous~~ self -pollinating true diploid (2n= 2x=14) ~~food~~ grain legume crop having genome size of 4063 Mb~~p/C genome size~~ [1]. The ~~domesticated~~ cultivated lentil species (*Lens* *culinaris*) encompasses two groups, identified established on the basis of distinct ~~phenotypic~~ morphological characters, the small-seeded (*microsperma*) and large-seeded (*macrosperma*) [2]. ~~Ferguson et al. [2] reported that~~ *~~L. odemensis~~* ~~and~~ *~~L. tomentosus~~* ~~established as subspecies of cultivated taxa, which manifested by the morphological as well as DNA markers studies. The production and productivity of lentil has enhanced increased from an average yield of 565 kg ha~~^~~-1~~^ ~~in 1961-63 to 940 kg ha~~^~~-1~~^ ~~during 2014-2015 [3]. Despite the tremendous increaseimprovement, the current lentil yield is much below as compared to other pulses. In India, most of the lentil varieties, which were developed through pure line selection resulted demonstrated theirshowed low yield potential base. In cultivated lentil varieties, the yield limiting factors are lack of seedling vigour, very high rate flower drop and low pod setting, lack of lodging resistance and exposure to major biotic and abiotic stresses [4]. The pedigree analysis of some important released varieties exhibited that a few donors have contributed substantially their blood into the background of those varieties [5]. Therefore, to attain further breakthrough for enhancing genetic gains, new target traits are needed to be identifiedy and introgressed them into cultivated gene pool for diversification ofwidening the genetic base of~~

cultigens. ~~On the other side, c~~Crop wild relatives (CWRs) are an invaluable ~~and always been taken as~~ ~~a~~ reservoir of productivity enhancement related characters~~, their adaptation~~having resilience to climate change and are ~~also providing researchers with desired~~source of novel traits~~gene sources~~ [6-7]. ~~To~~ ~~accomplish these challenges, all available~~The global wild lentil germplasm ~~was~~ introduced from the International Centre for Agricultural Research in Dry Areas (ICARDA), ~~which~~ was ~~further~~ multiplied, characterized and preliminary evaluated for various target traits [8]. The promising 96 wild lentil accessions selected from 405 global wild accessions were validated under multilocational testing for identifying stable donors against the target traits. ~~However, the promising 96 wild lentil accessions~~ ~~selected from 405 global wild accessions were further validated under multilocational testing for identifying stable donors against the target traits. Keeping above constraints in view, tThe present study was, therefore, undertaken (1) to evaluate promising wild lentil promising accessions for various target agronomical characters and major biotic stresses and (2) to identify the potential accessions (gene sources) carrying important traits for enhancing genetic gains of cultivated gene pool. The promising 96 wild lentil accessions selected from 405 global wild accessions were validated under multilocational testing for identifying stable donors against the target traits.~~

**Materials and methods**

**Plant materials**

The experimental genetic material comprised of 96 wild lentil accessions ~~The study was undertaken in~~ ~~96 wild lentil promising accessions~~ (Table 1) selected from 405 global wild ~~gene pool~~collections ~~(Singh et al. 2014)~~[8] consisting ~~24~~ of *L. orientalis* (24), ~~16 of~~ *L. odemensis* (16), ~~8 of~~ *L. tomentosus*

(8), ~~17 of~~ *L. nigricans* (17), ~~24 of~~ *L. ervoides* (24), ~~5 of~~ *L. lamottei* (5) and ~~2 of~~ *L. culinaris* (2) along with two check varieties (Precoz, and L830). These were evaluated under multi-location and multi-season ~~testing~~performance for target characters viz; earliness, pod number, seed yield and resistance against~~t to~~ rust, powdery mildew and Fusarium wilt under ~~hot spots~~field and controlled screening conditions. The original identity of these accessions was same as ~~also~~ mentioned by the Biodiversity

and Integrated Gene Management Unit (BIGM) at ICARDA.

**Agronomic evaluation**

All 96 wild lentil accessions were evaluated under two agro-ecological locations viz; Experimental Farm of NBPGR Regional Station, Shimla (28^0^ 35’ N, 70^0^18’E, altitude 2276 m amsl) during winter

**Formatted:** Font: Not Italic

season of 2014-2015, 2015-2016 and 2016-2017; and CSKHPKV~~,~~ Mountain Agricultural Research and Extension Centre, Sangla (31^0^ 55’ N, 77^0^ 00 50’ E altitude 3450 m amsl) during summer season of 2015, 2016 and 2017. Each entry was replicated thrice in 3 m long row and 35 cm apart. The observations were recorded against important agro-morphological characters in both ~~the~~ locations. Further, numerical data ~~were~~ was subjected to statistical analysis using SAS software [9]. The coefficient of variation for various characters was determined using the formulae of Burton [10] and broad sense heritability was also calculated as per the method suggested by Lush [11]. ~~However,~~The expected genetic advance was ~~also done as~~performed as per the procedure of Johnson et al. [12].

**Phenotypic diversity and association study**

The phenotypic diversity analysis was carried out using quantitative data of 96 promising accessions of wild lentil. Euclidean distances were calculated using quantitative data ~~and~~ which was used to establish relationship ~~among~~ between interspecific accessions. DARwin 5.0 software of Perrier et al.

[13] was used for hierarchical clustering of accessions using UPGMA mode based upon Jaccard’s

coefficient. Correlation among various parameters was determined using SAS software [9].

**Screening against rust, powdery mildew and fusarium wilt**

**Rust (*Uromyces*** fabae **(Grev.) Fuckel)**

~~This~~ The experiment was conducted at ~~Chaudhary Sarwan Kumar Himachal Pradesh~~CSKHPKV ~~Agricultural University~~ Research and Extension Centre, (CSKHPAUREC) Dhaulakuan ~~located at~~ (30^0^ 49´ N, 77^0^ 59´E, altitude 468 m amsl~~, longitude 30~~^~~0~~^ 49´ N, 77^~~0~~^ 59´E) during winter season of 2014-2015, 2015-2016 and 2016-2017. All the test accessions and rust susceptible check ‘var. L830’ were sown in plastic pots (20 cm diameter) filled with a mixture of top field soil and farm yard manure (10:1) in two replications.~~All accessions were sown under plastic pots filled with a mixture of top field~~ ~~soil and farm yard manure (10:1) replicated twice.~~ The pots with plants of wild accessions were shifted in the field sown with rust susceptible varieties, K 75 and L 830. ~~after 60 days of sowing~~. The test accessions were frequently inoculated by spraying ~~of~~ uerdo-acieosporic suspension (1x10^6^ spores/ml) of local isolate of *U. viciae fabae*. The ~~observations~~ data ~~were~~were recorded on terminal disease reaction using 1–9 rating scales ~~on terminal disease reaction~~ at vegetative and reproductive stages as followed by Mayee and Datar [14].

**Powdery mildew (*Erysiphe* *~~polygoni~~* ~~DC~~**~~.~~*trifolii*)

Another experiment was undertaken on ~~screening of wild lentil accessions against powdery mildew~~ at CSKHPKV Research and Extension Centre,~~CSKHPAUREC~~ Dhaulakuan during winter season of 2014-2015, 2015-2016 and 2016-2017. The plants of test accessions were raised in plastic pots in a mixture of soil and farmyard manure mixture (10:1) ~~and were continued to grow~~ ~~in the~~under green house conditions. The disease symptoms started developing at flower initiation stage and susceptible checks developed disease early and rapidly~~fastly and earlier~~. The heavily infected plants were shaken with the wooden stick to dislodge the conidia which dispersed and infected the healthy plant parts of the test wild accessions as was also ~~The pots with heavily infected susceptible plants were transferred~~ ~~near to test entries grown in the green house also and the infected plants were shaken well over the test wild accessions to disperse conidia as also suggested by Tiwari et al. [15]. The full infestation of susceptible accessions was observed near green pod stage and observations on terminal disease reaction were recorded at fourdifferent intervals using 1-9 rating scales [15].~~

**Fusarium wilt (*Fusarium oxysporum f. sp. lentis* (Vasd. Srin.) Gord)**

All 96 wild lentil accessions were screened against Fusarium wilt ~~resistance and the experiment was~~ ~~undertaken in a well maintained wilt sick plot~~ at Rafi Ahmad Kidwai Agriculture College, Sehore,

Madhya Pradesh (23^0^ 12’ N, 77^0^ 05’ E, altitude 502 m amsl) ~~India~~ during winter season of 2014-2015 and 2015-2016. For screening of wild lentil accessions, ~~following methods were followed as~~ ~~suggested~~protocol was standardized as per the method described by Bayaa and Erskine [16-19]. The performance of disease pressure was compared with the resistant (PL639) and susceptible (L-9-12) checks~~,~~ which were repeated ~~every~~ after 15 accessions of each replication. The data ~~were~~ was taken using 1-9 rating scales as followed by Bayaa et al. [17, 19].

**Results**

**Frequency distribution of quantitative characters**

The analysis of variance showed that the studied wild lentil genotypes differed significantly in terms of seed yield per plant and its important component characters ~~The results revealed wide range of~~ ~~variation as evident from the analysis of variance for different agro-morphological characters (significant at p=0.05) and it was furtherwere manifested by the range, mean and coefficient of~~

**Formatted:** Font: Italic

variation for all the metric characters assessed. However, the frequency distribution of important quantitative characters depicted in ~~f~~Fig~~ure~~. 1 exhibited that a wide range of variation was ~~assessed~~observed in plant height, number of branches plant^-1^, number of pods plant^-1^ and seed yield plant^-1^. In plant height, all 96 accessions were grouped into different classes (Fig. 1) and only two accessions ILWL15 (63.50 cm) and ILWL19 (66.50 cm) of *L. nigricans* were reported for maximum height. Likewise, ~~mean~~ number of branches plant^-1^ was highest in accession ILWL 418 (52 branches) of *L. ervoides* and ILWL 480 (37 branches) of *L. orientalis*. The number of pods plant^-1^ ranged from 3 pods plant^-1^ to 1167 pods plant^-1^ and only two accessions ILWL 418 (1002 pods) of *L. ervoides* and ILWL 18 (1167 pods) of *L. nigricans* revealed maximum number. The character seed yield plant^-1^ also showed variation and only two accessions ILWL 18 (13.60g) and ILWL 19 (12.55g) of *L. nigricans* ~~found~~ showed maximum seed yield palnt^-1^.

**Diversity analysis**

Wild lentil accessions formed two major clusters I and II in hierarchical clustering as shown in Fig. 2. Major cluster I grouped only three accessions including ILWL418 (*L. ervoides*) from Syria~~,~~; ILWL18 and ILWL19 (*L. nigricans*) from France and Spain, respectively. Cluster II ~~inhabited~~ inhabiting all remaining accessions was further divided into A and B clusters. Bifurcation of Cluster A resulted into two sub clusters AI and AII, ~~two sub clusters~~, where sub cluster AI was occupied by *L. ervoides* accessions ILWL58 and ILWL292*~~,~~* ~~both belonging to~~ from Turkey~~,~~; *L. nigricans* accessions ILWL16 from Alpes Cote d’ Azur and ILWL15 from France*~~,~~*; and ILWL14 and ILWL15 of *L. lamottei~~,~~* ~~both~~ from France. Sub cluster AII grouped ILWL7 and ILWL8 of *L. orientalis* ~~form~~ from Turkey;~~,~~ ILWL51, ILWL60 and ILWL65 of *L. ervoides* including first from Montenegro and ~~latter~~ other two from Turkey~~,~~; and ILWL20 of *L. odemensis* from Palestine. Cluster B was occupied by two sub clusters BI and BII, each of which were further divided into two groups a, b and c, d, respectively. Group d further formed outgroups (d1 and d2) and finally clutches (d2a and d2b). As Cluster II grouped sub-clusters were occupied by accessions from different wild lentil species belonging to different countries of origin, ~~was found highly diverse~~they showed highest diversity (Supplementary Table 1). Euclidean dissimilarity matrices for core set accessions varied from 6.01 between ILWL429 (*L. lamottei*) from Spain and ILWL90 (*L. tomentosus*) from Turkey to 2156.52 between ILWL18 (*L.* *nigricans*) from France and ILWL476 (*L. orientalis*) from Turkey ~~as shown in s~~(Supplementary ~~t~~Table 1). First two principal coordinates in factorial analysis~~,~~ explained 99.07 %~~,~~ and 0.52 % variability,

respectively. In both type of groupings, the role of geographical or country of origin of accessions was not observed.

**Correlations among different parameters**

The correlation coefficient among different agro-morphological traits of wild lentil accessions is shown in ~~t~~Table 2. Correlation indices highlighted a number of significant ~~(both +ve and -ve)~~ inter-relationships (both +ve and -ve) among studied traits. In correlation studies, when observations ~~are~~ were large in number, many correlations become significant due to increase in test power. So in the present study, correlations which had >0.4 r-values were considered. Significant positive correlation was observed between days to flowering and maturity. Similarly, significant positive correlations were obtained between seed yield plant^-1^, biological yield plant^-1^ and harvest index. These traits also showed positive correlations with plant height (r^2^ <4.0 in case of biological yield plant^-1^), number of branches plant^-1^ (r^2^ <4.0 in case of harvest index), number of pods plant^-1^ and seeds plant^-1^. Seeds plant^-1^ showed significant correlation with plant height, branches plant^-1^ and pods plant^-1^. The number of pods plant^-1^ exhibited significant positive correlation with branches plant^-1^. Significant negative correlation was observed between days to flowering and seed yield plant^-1^, seeds plant^-1^, pods plant^-1^, and harvest index.

**Principle component analysis**

The variability pattern among wild lentil accessions and the contribution of different traits towards these patterns were determined using Principal Component Analysis (PCA). Maximum variability (66.31%) was explained by only three components with Eigen value >1.0 (Table 3). Of the total variation, 42.37% was exhibited by PC1, and the contributing traits with high coefficients included seed yield plant^-1^, seeds plant^-1^, pods plant^-1^, harvest index and biological yield plant^-1^. Similarly, 15.04% variability was explained by PC2, and the contributing traits with high coefficients included days to maturity, days to flowering, rust incidence and plant height. Similarly, PC3 contributed 8.89% of overall variability, and the traits contributing to diversity included 100- seed weight, Fusarium wilt and Powdery mildew incidence. Fig. 3a and 3b shows the two dimensional variability pattern and contributing traits towards variability, respectively.

**Screening against major biotic stresses**

**Rust resistance (*Uromyces fabae* (Grev.) Fuckel)**

Majority of wild lentil accessions were resistant and moderately resistant against rust under controlled artificial inoculation conditions. Accessions ILWL90, ILWL195, ILWL198 and ILWL480 (*L.* *tomentosus*); ILWL230, 349, 476 (*L. orientalis*); ILWL203, 235 and 357 (*L. odemensis*); ILWL60, 204, 292, 414 and ~~ILWL~~442 (*L. ervoides*); and ILWL16, 18, 37, 460 and EC718266 (*L. nigricans*) were identified as resistant (Table 4 ~~&~~ and 5). The remaining accessions were scored as moderately resistant, susceptible and some of them were highly susceptible against the pathogen. The distribution of accessions into various reaction categories based on disease score is presented in ~~f~~Fig~~ure~~. 4.

**Powdery mildew resistance (*Erysiphe* *~~polygoni DC~~trifolii*)**

Out of 24 accessions of *L. orientalis*, ILWL 230 and ILWL 476 were found highly resistant.~~,~~ ~~l~~Likewise, in *L. odemensis*, accessions ILWL 39, ILWL 203, and IG 136788; ILWL 198 and ILWL 480 of *L. tomentosus* were also ~~reported~~ found to be highly resistant (Table 4 ~~&~~ and 5). However, accessions ILWL 51, ILWL 401, ILWL 418 and ILWL 441 of *L. ervoides*; ILWL9, ILWL22, ILWL34, ILWL37 and ILWL191 of *L. nigricans*; and ILWL 29 of *L. lamottei* with disease reaction 2 were found to be resistant. The remaining accessions were either moderately resistant or susceptible against the pathogen (Fig. 5).

**Fusarium wilt resistance (*Fusarium oxysporum f. sp. lentis* (Vasd. Srin.) Gord)**

The wild lentil accessions, which showed resistant disease reaction to Fusarium wilt included ILWL7, ILWL96, ILWL117, ILWL227, ILWL246, ILWL344 and ILWL359 (*L. orientalis*); ILWL15 and ILWL39 (*L. odemensis*); ILWL199 and ILWL 308 (*L. tomentosus*)*.* Accessions ILWL58, ILWL398, ILWL414, IG136626 (*L. ervoides*); ILWL17, ILWL19, ILWL23, ILWL34, ILWL38 and ILWL474 (*L. nigricans*) and ILWL20 and ILWL430 (*L. lamottei*) were found resistant against the wilt (Table 4

- and 5). Some accessions were moderately resistant and the remaining was susceptible against the pathogen ~~is~~ as depicted in ~~f~~Fig~~ure~~. 6.

**Discussion**

The precise evaluation of crop wild relatives is pre-requisite ~~with a view~~ to identify target traits of interest followed by their introgression into the background of cultivated varieties for enhancing genetic gains [20-22]. ~~We also found a~~A wide range of variation in wild lentil accessions was

**Formatted:** Font: Italic

observed against the target traits viz~~;~~. earliness, high pod number and seed yield as also ~~manifested~~ shown by the coefficient of variation, thereby suggesting diverse genetic makeup and geographical origins of wild lentil germplasm included in the present study. These promising accessions belonging to different taxa and ecological niches could be ~~a~~ useful resource for enhancing genetic gains of cultivated varieties. Accessions ILWL 18 and ILWL 19 of *L. nigricans* were found promising for high seed yield plant^-1^, suggesting their broader genetic base as also reflected in clustering pattern of wild accessions based on quantitative data analysis. Euclidean dissimilarity matrices of wild lentil accessions ranged from 6.01 to 2156.52, which revealed very high variability among interspecific lentil accessions. The observed variability could be due to the presence of diverse origins of these wild taxa. Among the different country of origin~~s~~, accessions from Turkey ~~region~~ showed maximum variability for the studied traits suggesting more exploration and collection of lentil germplasm. Viera et al. [23] characterized wheat germplasm based on phenotypic traits and obtained distances up to 196.61. The report of existing variability among germplasm accessions provides an idea about the expected heterosis for starting breeding ~~progarmme~~programme. A broad range of Euclidean distances obtained in the present study shows the importance of studied wild lentil accessions as a source of diverse heterotic material [24]. However, the diversity pattern further suggested that no role was played by the geographical distributions of global accessions in their grouping. This grouping pattern can be described on the basis of different genetic constitution of accessions. Further, it might be due to continuous gene flow occurrence among wild lentil taxa. No role of geographical distributions was ~~also~~ observed in grouping pattern of accessions clustered on the basis of morphological, biochemical and molecular markers in guar [25]. Similar observation in wild lentil accessions was also reported by Singh et al. [8] and Kumar et al. [26]. Further, significant positive correlation of seed yield plant^-1^ with seeds plant^-1^, pods plant^-1^, branches plant^-1^ was observed in lentil accessions and similar correlations have also been reported by ~~earlier~~ other workers [27-29]. They reported that seeds plant^-1^, biological yield plant^-1^, pods plant^-1^, and harvest index are the important yield component traits in lentil contribute~~ing~~ towards yield enhancement. Such correlations are useful for lentil genetic improvement and breeding ~~as well~~programmes. These observations ~~are~~ were found to be in accordance with the results of Toklu et al. [29] and Abo-Hegazy et al. [30]. The traits which contributed heavily towards variability included seed yield plant^-1^, seeds pod^-1^, pods plant^-1^, biological yield plant^-1^ and harvest index. These findings highlighted the significance of yield related component characters generating substantial variations in wild lentil accessions.

Furthermore, wild taxa of lentil are known to be resistant against major biotic and abiotic stresses [8]. They ~~suggested that wild lentil accessions~~ are the carrier of important characters and ~~these~~ ~~may~~ have potential value in diversification of cultivated gene pool ~~diversification~~ for enhancing genetic gains. In the promising experiments on screening of wild lentil accessions against major biotic stresses, development of severe disease symptoms on the susceptible cultivars were clearly evident in the real field conditions, indicating the effectiveness of inoculation procedures. Some wild lentil accessions revealed resistant disease reaction against more than one disease (Table 5) ~~i.e.~~viz. accessions ILWL230 and ILWL476 of *L. orientalis* (rust and powdery mildew); accessions ILWL9 and ILWL37 of *L. nigricans* (rust and powdery mildew); accession IG136639 of *L. ervoides* (powdery mildew and Fusarium wilt) and accession ILWL308 of *L. tomentosus* (rust and Fusarium wilt). It is important to mention that the northwestern part has been the traditional lentil cultivation area in India. But in late 1980s, among various factors~~,~~ including the epidemics of rust, powdery mildew and Fusarium wilt has resulted in drastic reduction of lentil area and production as well. ~~Because, most of~~ ~~the lentil varieties grown in these regions became susceptible against prevailing diseases.~~ In this context, gene sources identified against rust, powdery mildew and *Fusarium* wilt ~~resistant gene~~ ~~sources identified~~ in the present study can prove to be useful donors which could be ~~exploiting~~ exploited through pre-breeding with the aim to develop usable germplasm for ~~further~~ ~~breeding~~developing resistant lentil varieties, especially for the epidemiologically important Indian conditions. Furthermore, the development of lentil cultivars with combined resistance against major biotic stresses may help in the development of appropriate management strategies in future in conjunction with allelism studies to identify and pyramid novel genes for resistance breeding of lentil.

**Conclusions**

During the past few years, little efforts were made on evaluation of ~~small number of~~ wild lentil genetic resources in India. However, this is the first attempt on characterization and evaluation of all available global wild lentil germplasm against the target traits of interest under diverse agro-ecological conditions. Further, the study has also helped in identifying the confirmed and stable gene sources (donors) across intra and interspecific accessions viz~~;~~. ILWL203 (EC789113) of *L. odemensis* for rust and high pod number; ILWL230 (EC718515), ILWL476 (EC718605) of *L. orientalis* for rust and powdery mildew; ILWL191 (EC718682), ILWL9, and ILWL37 of *L. nigricans* for rust and powdery mildew, IG136639 of *L. ervoides* for powdery mildew and Fusarium wilt and ILWL308 (EC728782) of *L. tomentosus* for rust and Fusarium wilt. These gene sources could be taken in lentil wide

hybridization programme for enhancing genetic gains of cultivated varieties and could also be shared

among lentil breeders/researchers in the country or elsewhere under Standard Material Transfer

Agreement (SMTA) for strengthening ~~their~~ the on-going lentil genetic improvement programmes.

**Acknowledgements**

We thank the Head of ~~g~~Gene bank at ICARDA, Aleppo Syria for providing all available wild lentil

accessions to ~~India~~us. The authors also thank the Department of Agriculture Cooperation and Farmers

Welfare, Govt. of India for grant in aid to accomplish the proposed objectives.

**References**

1. Arumuganathan K., Earle ED. Nuclear DNA content of some important plant species. Plant Mol. Biol Rep*.*1991; 9: 208–218.
2. Ferguson ME, Maxted N, Slageren MV, Robertson LD. A re-assessment of the taxonomy of Lens. Bot. J. Linn. Soc. 2000; 133: 41–59.
3. Food and Agriculture Organization (FAO) FAO Statistical database of the United Nations, Food and Agriculture Organization Rome; 2015.
4. Erskine W, Muehlbauer FJ, Sarker A, Sharma B. An Introduction, In: W. Erskine, F.J.

Muehlbauer, A. Sarker and B. Sharma: The lentil, botany, production and uses. CAB International; 2009 p.1-3.

5. Kumar S, Gupta S, Chandra S, Singh BB. How wide is the genetic base of pulse crops, Pulses in New Perspective In: Ali M, Singh BB, Kumar S, Dhar V eds. Indian Society of Pulses Research and Development, Indian Institute of Pulses Research, Kanpur India; 2003.
6. Maxted N, Ford BV, Kell SP, Iriondo JM, Dulloo ME. Crop wild relative conservation and use, Wallingford, UK, CABI publishing; 2008.
7. Lane A, Jarvis A. Changes in climate will modify the geography of crop suitability: Agricultural biodiversity can help with adaptation. Climate Proofing Innovation for Poverty Reduction and Food Security, ICRISAT, Patancheru; 2007.
8. Singh M, Bisht IS, Kumar S, Dutta M, Bansal KC, Sarker A, Amri A, Kumar S, Datta SK. Global wild annual Lens collection: a potential resource for lentil genetic base broadening and yield enhancement. PLoS ONE, (2014) 10.1371/journal.pone.0107781
9. SAS. Sas Institute Inc. Cary, NC, SAS Institute; 2011.
10. Burton GW. Quantitative inheritance in grasses, in Proceedings of the 454 International Grassland Congress, State College, PA: Pennsylvania State College; 1952.
11. Lush JL. Intrusive collection of regression of offspring on dams as a method 501 of estimating heritability of characters. Proc. Am. Soc. Anim. Prod. 1940; 33: 293–301.
12. Johnson HW, Robinson HF, Comstock RE. Estimates of genetic and environmental variability in Soybean. Agron. 1955; J. 47: 314–318.
13. Perrier X, Flori A, Bonnot F. Data analysis methods, In: Hamon, P., Seguin, M., Perrier, X., Glaszmann, J.C. Ed., Genetic diversity of cultivated tropical plants. Enfield, Science Publishers Montpellier; 2003.

14. Mayee CD, Datar VV. Phytopathometry. Technical Bulletin-1, Marathwada Agricultural University, Parbhani; 1986.
15. Tiwari KR, Penner GA, Warkentin TD, Rashid KY. Pathogenic variation in Erisiphe polygoni, the casual organism of powdery mildew of lentil. Can. J. Plant Pathol. 1997; 19: 267–271.
16. Bayaa B, Erskine W. A screening technique for resistance to vascular wilt in lentil. Arab J. Plant Prot. 1990; 8: 30–33.
17. Bayaa B, Erskine W, Hamdi A. Evaluation of wilt lentil collection for resistance to vascular wilt. Genet. Resour. Crop Evol. 1995; 42: 231–235.
18. Bayaa B, Erskine W, Singh M. Screening lentil for resistance to Fusarium wilt: methodology

and sources of resistance. Euphytica. 1997; 98: 69–74.

19. Eujayl I, Erskine W, Bayaa B, Baum M, Pehu E. Fusarium vascular wilt in lentil: inheritance

and identification of DNA markers for resistance. Plant Breed. 1998; 117: 497–499.

20. Duvick DN. Genetic diversity in major farm crops on the farm and in reverse. Eco. Bot. 1984;
    38: 151–178.
21. Lazaro A, Ruiz M, Rosa L, Martin I. Relationship between agro-morphological characters and climate parameters in Spanish landraces of lentil. Genet Resour Crop Evol. 2001; 48: 239-249.
22. Naghavi MR, Jahansouz MR. Variation in the agronomic and morphological traits of lentil accessions. J. Integ. Plant Biol. 2005; 47: 375–379.
23. Vieira EA, Carvalho FIF, Bertan de I, Kopp M, Zimmer PD. Association between genetic distances in wheat as estimated by AFLP and morphological markers. Genet. Mol. Biol. 2007;
    30: 392–399.
24. Gupta SK, Nepolean T, Shaikh CG, Rai K, Hash CT, Das RR, Rathore A. Phenotypic and molecular diversity-based prediction of heterosis in pearl millet (Pennisetum glaucum L. (R.) Br.).The Crop Journal. 2018; 6: 271-281.
25. Kumar S, Joshi UN, Singh V, Singh JV, Saini ML. Characterization of released and elite genotypes of guar from Indian proves unrelated to geographical origin. Genet Resour Crop Evol. 2013; 60: 2017-2032.
26. Kumar S, Choudhary AK, Rana KS Sarker A, Singh M. Biofortification potential of global

wild annual lentil core collection. PLOS ONE. 2018; [https://doi.org/10.1371/journal.pone.0191122.](https://doi.org/10.1371/journal.pone.0191122)

27. Luthra SK, Sharma PC. Correlation and path analysis in lentil. Lens New Letter. 1990; 7: 5-8.
28. Sarwar G, Abbas G, Asghar MJ. Quantitative analysis of yield related traits in lentil. J. Agric. Res. 2013; 51: 239-246.
29. Toklu F, Ozkan H, Karakoy T, Coyne C. Evaluation of advanced lentil lines for diversity in seed mineral concentration, grain yield and yield components. J Agric. Sci. 2015; 23: 213-222.
30. Abo-Hegazy SRE, Selim T, El-Emam EAA. Correlation and path co-efficient analysis of yield and some yield components in lentil. Egyptian J. Pl. Breed. 2012; 16: 147-159.
31. Wong MM, Gujaria N, Ramsay L, Yuan HY, Caron C, Diapari M, Vanderberg A, Bett KE. Classification and characterization of species within the genus *Lens* using genotyping-by-sequencing. PLoS ONE. 2015; 10(3) e0122025.

**Table 1: List of taxa wise wild lentil accessions along with their country origin**

| **S.** | **Taxa/accession** | **Origin** | **S. No.** | **Taxa/accession** | **Country origin** |
| --- | --- | --- | --- | --- | --- |
| **No** |  |  |  |  |  |
|  | ***L. orientalis*** |  |  | ***L. lamottei*** |  |
| 1 | ILWL 7 (EC718234) | Turkey | 50 | ILWL 14 (EC718236) | France |

| 2 | ILWL 8 | (EC718235) | Turkey | 51 | ILWL 15 | (EC718238) | France |
| --- | --- | --- | --- | --- | --- | --- | --- |
| 3 | ILWL 75 (EC718456) | | Israel | 52 | ILWL 29 | (EC718251) | Spain |
| 4 | ILWL 89 | (EC718470) | Turkey | 53 | ILWL 429 (EC718686) | | Spain |
| 5 | ILWL 95 | (EC718475) | Turkey | 54 | EC718692 |  | France |
| 6 | ILWL 96 | (EC718476) | Turkey |  | ***L.* o*demensis*** | |  |
| 7 | ILWL 101 | (EC718479) | Turkey | 55 | ILWL 20 | (EC718243) | Palestine |
| 8 | ILWL 117 (EC718488) | | Syria | 56 | ILWL 35 | (EC718276) | Turkey |
| 9 | ILWL 124 (EC718490) | | Syria | 57 | ILWL 39 | (EC718277) | Palestine |
| 10 | ILWL 181 (EC718491) | | Syria | 58 | ILWL 165 (EC718281) | | Syria |
| 11 | ILWL 227 (EC718513) | | Syria | 59 | ILWL 166 (EC718282) | | Syria |
| 12 | ILWL 230 (EC718515) | | Syria | 60 | ILWL 167 (EC718283) | | Syria |
| 13 | ILWL 243 (EC718519) | | Syria | 61 | ILWL 203 (EC789113) | | Syria |
| 14 | ILWL 246 (EC718521) | | Syria | 62 | ILWL 235 (EC718291) | | Syria |
| 15 | ILWL 278 (EC718529) | | Turkey | 63 | ILWL 320 (EC718295) | | Turkey |
| 16 | ILWL 330 (EC718548) | | Syria | 64 | ILWL 357 (EC718297) | | Syria |
| 17 | ILWL 343 (EC718554) | | Syria | 65 | ILWL 361 (EC718298) | | Syria |
| 18 | ILWL 344 (EC718555) | | Syria | 66 | ILWL 409 (EC718301) | | Syria |
| 19 | ILWL 349 ( EC718560) | | Syria | 67 | ILWL 436 (EC718302) | | Turkey |
| 20 | ILWL 359 (EC718566) | | Syria | 68 | ILWL 438 (EC718303) | | Turkey |
| 21 | ILWL 384 (EC718585) | | Tajikistan | 69 | EC718311 |  | Israel |
| 22 | ILWL 443 (EC718596) | | Turkey | 70 | EC718694 |  | Syria |
| 23 | ILWL 476 (EC718605) | | Turkey |  | ***L. ervoides*** |  |  |
| 24 | ILWL 480 (EC718609) | | Syria | 71 | EC718692 |  | Spain |
|  | ***L. nigricans*** | |  | 72 | ILWL 43 | (EC718316) | Croatia |
| 25 | ILWL 9 | (EC718236) | Syria | 73 | ILWL 50 | (EC718321) | Croatia |
| 26 | ILWL 14 | (EC718267) | Syria | 74 | ILWL 51 | (EC718322) | Montenegro |
| 27 | ILWL 15 | (EC718238) | France | 75 | ILWL 58 | (EC718329) | Turkey |
| 28 | ILWL16 | (EC718239) | Alpes-Cote |  |  |  |  |
|  |  |  | d'Azur | 76 | ILWL 60 | (EC718331) | Turkey |
| 29 | ILWL 18 | (EC718241) | France | 77 | ILWL 61 | (EC718332) | Turkey |
| 30 | ILWL 19 | (EC718242) | Spain | 78 | ILWL 63 | (EC718333) | Turkey |
| 31 | ILWL 22 | (EC718245) | Italy | 79 | ILWL 65 | (EC718335) | Turkey |
| 32 | ILWL 31 | (EC718254) | Spain | 80 | ILWL 66 | (EC718336) | Turkey |
| 33 | ILWL 34 | (EC718256) | Ukraine | 81 | ILWL 204 (EC718360) | | Turkey |
| 34 | ILWL 37 | (EC718257) | Turkey | 82 | ILWL 234 (EC718362) | | Syria |
| 35 | ILWL 38 | (EC718258) | Turkey | 83 | ILWL 269 (EC718370) | | Turkey |
| 36 | ILWL 191 (EC718682) | | Croatia | 84 | ILWL 276 (EC718374) | | Turkey |
| 37 | ILWL 460 (EC718262) | | Turkey | 85 | ILWL 292 (EC718378) | | Turkey |
| 38 | EC718266 | | Italy | 86 | ILWL 321 (EC718383) | | Turkey |
| 39 | EC718270 | | Croatia | 87 | ILWL 398 (EC718401) | | Lebanon |
| 40 | EC718273 | | Spain | 88 | ILWL 401 (EC718404) | | Lebanon |
| 41 | EC718275 | | Turkey | 89 | ILWL 408 (EC718407) | | Syria |
|  | ***L. tomentosus*** | |  | 90 | ILWL 414 (EC718411) | | Syria |
| 42 | ILWL 90 | (EC718471) | Turkey | 91 | ILWL 418 (EC718413) | | Syria |
| 43 | ILWL 195 (EC718682) | | Syria | 92 | ILWL 441 (EC718417) | | Turkey |
| 44 | ILWL 198 (EC718685) | | Syria | 93 | ILWL 442 (EC718418) | | Turkey |
| 45 | ILWL 199 (EC718686) | | Syria | 94 | EC718439 |  | Israel |
| 46 | ILWL 305 | (EC728782) | Turkey |  | ***L. culinaris*** | |  |
| 47 | ILWL 308 | (EC728782) | Turkey | 95 | ILL 8006 |  | Syria |
| 48 | ILWL 480 | (EC741251) | Syria | 96 | ILL 10829 |  | Syria |
| 49 | EC718673 | | Syria |  |  |  |  |

**Table2: Correlations among various agro-morphological traits of wild lentil accessions**

| **Traits** | **DF** | **DM** | **PH** | **NBPP** | **NPPP** | **NSPPL** | **NSPPD** | **SDWT** | **SYPP** | **BYPP** | **HI** | **RT** | **PM** | **FW** |
| --- | --- | --- | --- | --- | --- | --- | --- | --- | --- | --- | --- | --- | --- | --- |
| **DF** | 1.00 | .750(**) | -0.13 | -.219(*) | -.542(**) | -.534(**) | -0.16 | -0.06 | -.495(**) | - | -.543(**) | -0.15 | -0.06 | -0.15 |
|  |  |  |  |  |  |  |  |  |  | .311(**) |  |  |  |  |
|  |  |  |  |  |  |  |  |  |  |  |  |  |  |  |
| **DM** | .750(**) | 1.00 | 0.04 | -0.15 | -.346(**) | -.319(**) | -.256(*) | -0.09 | -.350(**) | -.235(*) | -.319(**) | -0.02 | 0.03 | -0.17 |
|  |  |  |  |  |  |  |  |  |  |  |  |  |  |  |
| **PH** | -0.13 | 0.04 | 1.00 | .242(*) | .360(**) | .411(**) | -0.11 | -0.15 | .468(**) | .344(**) | .438(**) | .342(**) | 0.15 | -0.14 |
|  |  |  |  |  |  |  |  |  |  |  |  |  |  |  |
| **NBPP** | -.219(*) | -0.15 | .242(*) | 1.00 | .544(**) | .536(**) | -.285(**) | -0.09 | .504(**) | .539(**) | .388(**) | .328(**) | .272(**) | 0.08 |
|  |  |  |  |  |  |  |  |  |  |  |  |  |  |  |
| **NPPP** | -.542(**) | -.346(**) | .360(**) | .544(**) | 1.00 | .987(**) | -0.15 | -0.09 | .928(**) | .833(**) | .738(**) | .367(**) | .290(**) | 0.03 |
|  |  |  |  |  |  |  |  |  |  |  |  |  |  |  |
| **NSPPL** | -.534(**) | -.319(**) | .411(**) | .536(**) | .987(**) | 1.00 | -0.18 | -0.08 | .956(**) | .820(**) | .788(**) | .308(**) | .322(**) | -0.02 |
|  |  |  |  |  |  |  |  |  |  |  |  |  |  |  |
| **NSPPD** | -0.16 | -.256(*) | -0.11 | -.285(**) | -0.15 | -0.18 | 1.00 | -0.12 | -.244(*) | -.247(*) | -0.16 | -.291(**) | -.224(*) | 0.13 |
|  |  |  |  |  |  |  |  |  |  |  |  |  |  |  |
| **SDWT** | -0.06 | -0.09 | -0.15 | -0.09 | -0.09 | -0.08 | -0.12 | 1.00 | 0.00 | -0.12 | 0.02 | -0.02 | 0.07 | 0.14 |
|  |  |  |  |  |  |  |  |  |  |  |  |  |  |  |
| **SYPP** | -.495(**) | -.350(**) | .468(**) | .504(**) | .928(**) | .956(**) | -.244(*) | 0.00 | 1.00 | .814(**) | .841(**) | .373(**) | .391(**) | 0.00 |
|  |  |  |  |  |  |  |  |  |  |  |  |  |  |  |
| **BYPP** | -.311(**) | -.235(*) | .344(**) | .539(**) | .833(**) | .820(**) | -.247(*) | -0.12 | .814(**) | 1.00 | .508(**) | .377(**) | .283(**) | -0.02 |
|  |  |  |  |  |  |  |  |  |  |  |  |  |  |  |
| **HI** | -.543(**) | -.319(**) | .438(**) | .388(**) | .738(**) | .788(**) | -0.16 | 0.02 | .841(**) | .508(**) | 1.00 | .312(**) | .347(**) | 0.00 |
|  |  |  |  |  |  |  |  |  |  |  |  |  |  |  |
| **Rust** | -0.15 | -0.02 | .342(**) | .328(**) | .367(**) | .308(**) | -.291(**) | -0.02 | .373(**) | .377(**) | .312(**) | 1.00 | .314(**) | -0.08 |
|  |  |  |  |  |  |  |  |  |  |  |  |  |  |  |
| **PM** | -0.06 | 0.03 | 0.15 | .272(**) | .290(**) | .322(**) | -.224(*) | 0.07 | .391(**) | .283(**) | .347(**) | .314(**) | 1.00 | -0.02 |
|  |  |  |  |  |  |  |  |  |  |  |  |  |  |  |
| **FW** | -0.15 | -0.17 | -0.14 | 0.08 | 0.03 | -0.02 | 0.13 | 0.14 | 0.00 | -0.02 | 0.00 | -0.08 | -0.02 | 1.00 |
|  |  |  |  |  |  |  |  |  |  |  |  |  |  |  |

*Correlation is significant at the 0.05 level, **Correlation is significant at the 0.01 level

DF, Days to flowering; DM, Days to maturity; PH, Plant height; NBPP, Number of branches plant^-1^; NPPP, Number of pods plant^-1^; NSPPL, Number of seeds plant^-1^; NSPPD, Number of seeds pod^-1^; SDWT, Seed weight; SYPP, Seed yield plant; BYPP, Biological yield plant; HI, Harvest index; RT, Rust; PM, Powdery mildew; FW, Fusarium wilt

**Table 3: Eigen vectors, eigen values, individual and cumulative percentages of variation explained by the first three principal components (PC) of wild lentil accessions**

| **Traits** | **Prin1** | **Prin2** | **Prin3** |
| --- | --- | --- | --- |
| Days to flowering | -0.25 | 0.49 | 0.00 |
| Days to maturity | -0.19 | 0.56 | -0.01 |
| Plant height (cm) | 0.20 | 0.22 | -0.30 |
| Number of branches plant^-1^ | 0.25 | 0.12 | 0.09 |
| Number of pods plant^-1^ | 0.39 | -0.05 | -0.06 |
| Number of seeds plant^-1^ | 0.39 | -0.02 | -0.06 |
| Number of seeds pod^-1^ | -0.10 | -0.41 | -0.37 |
| 100 -seed weight (g) | -0.02 | -0.09 | 0.71 |
| Seed yield plant^-1^ (g) | 0.40 | 0.04 | 0.02 |
| Biological yield plant^-1^ (g) | 0.34 | 0.10 | -0.06 |
| Harvest index (%) | 0.35 | -0.04 | 0.02 |
| Rust | 0.26 | 0.26 | 0.04 |
| Powdery mildew | 0.17 | 0.22 | 0.33 |
| Fusarium wilt | 0.00 | -0.26 | 0.37 |
| Eigen value | 5.93 | 2.11 | 1.24 |
| Percent | 42.37 | 15.04 | 8.89 |
| Cum. Percentage | 42.37 | 57.42 | 66.31 |

**Table: 4. Range of variation of wild lentil accessions against the pathogens of rust, powdery mildew and *Fusarium* wilt**

| **Species** |  | **Rust** |  |  | **Powdery mildew** |  |  | **Fusarium wilt** | |  |
| --- | --- | --- | --- | --- | --- | --- | --- | --- | --- | --- |
|  | **Range** | **Mean ±SE** | **CV (%)** | **Range** | **Mean ±SE** | **CV (%)** | **Range** | **Mean ±SE** |  | **CV (%)** |
| ***L. culinaris*** | 1-1 | 1.00±0.00 | 0.00 | 0-5 | 5.00±0.00 | 0.00 | 1-7 | 4.00±3.00 |  | 106.07 |
| ***L. orientalis*** | 1-9 | 3.41±0.70 | 84.23 | 0-5 | 3.56±0.35 | 42.29 | 1-9 | 4.44±0.74 |  | 70.40 |
| ***L. tomentosus*** | 1-5 | 1.86±0.59 | 84.73 | 0-5 | 2.29±0.75 | 86.45 | 1-9 | 5.00±1.15 |  | 61.10 |
| ***L. odemensis*** | 1-6 | 2.33±0.40 | 66.13 | 0-4 | 1.60±0.39 | 93.90 | 1-9 | 5.67±0.64 |  | 43.57 |
| ***L. ervoides*** | 1-8 | 1.95±0.42 | 92.93 | 0-5 | 1.90±0.45 | 105.12 | 1-9 | 5.18±0.72 |  | 65.14 |
| ***L. nigricans*** | 1-7 | 2.10±0.46 | 98.81 | 0-5 | 2.52±0.52 | 95.11 | 1-9 | 4.43±0.69 |  | 71.61 |
| ***L. lamottei*** | 1-7 | 3.43±0.97 | 75.04 | 0-5 | 3.29±0.75 | 60.14 | 1-9 | 4.43±1.04 |  | 62.33 |

**Table 5: Identification of donor accessions for their introgression to lentil genetic improvement against important agronomic and major biotic stress related traits**

| **Taxa/ trait** | **Accession** |  | **Gene pool** | **Country origin** |
| --- | --- | --- | --- | --- |
| **Agronomic** |  |  |  |  |
| *L. orientalis* | ILWL255, 476 ((High pods plant^-1^, shorter | internode) | Primary | Turkey, Syria |
| *L. odemensis* | ILWL203, 237 (High pods plant^-1^, shorter | internode) | Primary | Syria |
| *L. ervoides* | ILWL294, 321 ((High pods plant^-1^, shorter | internode) | Tertiary | Turkey, Syria |
| **Rust** |  |  |  |  |
| *L. orientalis* | ILWL230, 349, 466 |  | Primary | Turkey |
|  |  |  |  |  |
| *L. odemensis* | ILWL235, 357, ILWL203 |  | Primary | Syria |
| *L. tomentosus* | ILWL 90, 95,198, 308, 480 |  | Primary | Syria |
| *L. ervoides* | ILWL60, 92, 269, 292, 414, 419, IG136226 | | Tertiary | Turkey, Syria |
| *L. nigricans* | ILWL9, 17, 37, 111, 460, IG136639, IG136648 | | Quaternary |  |
| **Powdery mildew** |  |  |  |  |
| *L. orientalis* | ILWL 230, 466 |  | Primary | Syria |
| *L. odemensis* | ILWL 39, 196, IG136788 |  | Primary | Turkey, Syria |
| *L. tomentosus* | ILWL 198, 480 |  | Primary | Turkey, Syria |
| *L. ervoides* | ILWL51, 401, 418, 441 |  | Tertiary | Montenegro, Lebanon, |
|  |  |  |  | Syria, Turkey |
| *L. nigricans* | ILWL6, 9, 22, 34, 37, 111 |  | Quaternary | Turkey, Syria Italy, |
|  |  |  |  | Ukraine, Turkey, Turkey |
| *L. lamottei* | ILWL 29 |  | Secondary | Spain |
| **Fusarium wilt** |  |  |  |  |
| *L. orientalis* | ILWL7, 96, 117, 227, 246, 344, 359 |  | Primary | Turkey, Turkey, Syria, |
|  |  |  |  | Syria, Syria, Syria, Syria, |
| *L. odemensis* | ILWL15, 39 |  | Primary | France, Turkey |
| *L. tomentosus* | ILWL 199, 308 |  | Primary |  |
| *L. ervoides* | ILWL 58, 398, 414, IG136626 |  | Tertiary | Turkey , Lebanon, Syria |
|  |  |  |  | Israeli |
| *L. nigricans* | ILWL17, 19, 23, 34, 38, 474 |  | Quaternary | Spain, Italy, Ukraine, |
|  |  |  |  | Turkey, Turkey |
| *L. lamottei* | ILWL 20, 430 |  | Secondary | PAL, Spain, |

Gene pool classification of Wong et al. [31]

Figure	[Click here to access/download;Figure;Manuscript-figure-PLOS ONE.docx](https://www.editorialmanager.com/pone/download.aspx?id=25794310&guid=b61f8ecd-e55a-4bbc-9fc1-ab8289ec0efe&scheme=1)

**Fig:1 Frequency distribution of wild lentil accessions for important quantitative traits**

Cluster II

Cluster II

**Fig. 2: Hierarchical clustering of wild lentil accessions based on quantitative data analysis**

**Fig. 3: PCA analysis - (a) 2-D graph of first two principal components and (b) various traits contributing**

**to the variability**

**Fig. 4: Screening of wild lentil accessions against rust resistance**

**Fig. 5: Screening of wild lentil accessions against powdery mildew resistance**

**Fig. 6: Screening of wild lentil accessions against Fusarium wilt resistance**

Supporting Information

[Click here to access/download](https://www.editorialmanager.com/pone/download.aspx?id=25794311&guid=45c43980-546e-43b2-857f-ca40e87855db&scheme=1)

[**Supporting Information**](https://www.editorialmanager.com/pone/download.aspx?id=25794311&guid=45c43980-546e-43b2-857f-ca40e87855db&scheme=1)

[Supplementary table-1.docx](https://www.editorialmanager.com/pone/download.aspx?id=25794311&guid=45c43980-546e-43b2-857f-ca40e87855db&scheme=1)
