# Supplementary material for: Evaluation and identification of wild lentil accessions for enhancing genetic gains of cultivated varieties
Source: PLoS One. 2020 Mar 3;15(3):e0229554. doi: 10.1371/journal.pone.0229554 (PMC7053756; doi:10.1371/journal.pone.0229554)
Supplement: Attachment — Submitted filename: Reply to reviewer-comments.docx [file pone.0229554.s004.docx]

**Reply to reviewer’s comments and submission of revised version**

**Dear Academic Editor and Reviewers**

We tried our level best to improve the proposed research article on wild lentil and necessary changes were made in the revised text using track change mode.

**Reviewer 1**

Although as such no comments were mentioned separately, we have included all changes made in the manuscript as suggested and we really appreciate the changes suggested in the paper.

**Reviewer 2**

**Comment:** In plant materials, you mentioned check varieties: Precoz and L830. Kindly add their origin in table 1 and why did you choose those 2 varieties.

**Reply:** The country of origin for these two varieties has been mentioned in revised text in the Material and method section. As far as taking these varieties as checks are concerned, these lentil varieties are having very good agronomic base in majority of lentil growing areas in India.

**Comment:** Did K75 was used as check variety for Rust?

**Reply:** Yes for rust susceptibility

**Comment:** Did PL639 and L-9-12 were used as check varieties for Fusarium wilt? Kindly add their origins

**Reply:** Yes taken as checks and origins have been added in the modified text.

.
